# Phytochemical, biological, DFT, and molecular docking evaluation of *Euphorbia paralias*

**DOI:** 10.1038/s41598-025-02420-1

**Published:** 2025-05-23

**Authors:** Abdullah I. Kamel, Safa A. Badawy, Mamdouh Abdel-Mogib, Ahmed Ramadan El-Rokh

**Affiliations:** 1 Chemistry Department, Faculty of Science, New Mansoura University, Mansoura, Egypt; 2 Plant Protection Research Institute, Agricultural Research Center, Giza, 12618 Egypt; 3 Chemistry Department, Faculty of Science, Mansoura University, Mansoura, 35516 Egypt

**Keywords:** Natural products, *Euphorbia paralias*, Flavonoid glycosides, Bioactivity, Antibacterial activity, Insecticidal activity, Quantum chemical analysis (DFT), Molecular docking, Cheminformatics, Organic chemistry, Structure elucidation

## Abstract

This study aimed to bridge the knowledge gap in the unclear previous studies of the molecular processes that cause *the biological activities of Euphorbia paralias* by integrating phytochemical analysis with quantum chemical calculations and molecular docking investigations, providing unprecedented insights into the therapeutic potential of its chemical constituents. Seven important flavonoids were isolated and identified using spectroscopic techniques, and 34 and 13 additional compounds were identified via GC/MS analysis of the hexane and chloroform fractions, respectively. The crude methanol extract, some fractions, and isolated compounds were screened for antimicrobial activity against Gram-positive and Gram-negative bacteria. Among the tested constituents,* β*-sitosterol-3-*O-β*-d-glucoside **1**, kaempferol-3-*O-α*-d-arabinopyranoside **4**, and genistein-8-*β*-C-glucoside **6**, as well as the chloroform and ethyl acetate fractions, demonstrated notable broad-spectrum antibacterial activity. The insecticidal activities of the butanol fraction and a combination of genistein-4′-*O-β*-d-glucopyranoside **2** and quercetin-3-*O-β*-d-glucoside **3** significantly inhibited *Aphis gossypii* and *Amrasca biguttula*, with LC_50_ values of 397.39 ppm and 332.92 ppm, respectively. DFT calculations at the B3LYP/6-31G(d) level revealed that hirsutissimiside B **7** exhibited the lowest HOMO–LUMO gap (1.643 eV), highest dipole moment (7.562 Debye), and lowest chemical hardness (0.821 eV), suggesting enhanced chemical reactivity and bioactivity. Molecular docking simulations revealed the strong binding affinities of the active compounds to key microbial and insecticidal target proteins. The high degree of concordance between computational predictions and experimental bioactivity results reinforces the therapeutic potential of these natural products. These findings highlight the synergistic value of integrating quantum chemical calculations, molecular modeling, and biological assays to advance natural product-based drug discovery and pest control strategies.

## Introduction

*Euphorbia paralias*, a member of the Euphorbiaceae family, is a hardy perennial plant native to the Mediterranean region and is typically found on shingle beaches and sandy coasts. It can grow up to 70.0 cm in height and is known for its medicinal properties and ecological resilience^1,2^. Various *Euphorbia* species have been used in folk medicine to address ailments involving the liver, uterus, and stomach and to manage symptoms of asthma^3^. Additionally, other species within this genus have been reported to be used for treating intestinal parasites, gonorrhea, migraines^4^, inflammation, as local anesthetic^5^ and even cancer^6–12^. These diverse applications highlight the therapeutic potential of *Euphorbia* species. Phytochemical investigations of *E. paralias* have revealed a complex mixture of bioactive compounds, including flavonoids such as quercetin-3-arabinoside, kaempferol-3-(6″-(2‴-galloyl-glucopyranoside), and quercetin-3-*O-β*-d-glucoside^13^, hyperin^14^ as such as segetanin A and B, and tricyclic diterpenoids such as pre-segetanin^15–17^. Furthermore, the presence of tetracyclic diterpenes (ingenanes, jatrophanes^14^), pentacyclic triterpenoids (*β*-amyrin, betulinol, erythradiol), and sterols (campesterol, *β*-sitosterol–3-*O-*glucoside, sitosterol, stigmasterol, cholesterol)^13^ underscores the chemical diversity and potential pharmacological benefits of this species. The increasing resistance of microbes and pests to synthetic antimicrobial and insecticidal agents has intensified the search for natural alternatives with fewer side effects. Natural insecticides and antimicrobials derived from plants hold an intriguing place. In addition to their rapid environmental degradation, these compounds have a high safety rate for both humans and the environment. Insect resistance to synthetic pesticides is a result of their overuse, which also damages humans, animals, and plants. Plant-derived compounds are increasingly recognized as viable sources of such agents, particularly when supported by both traditional use and modern bioactivity screening. *Aphis gosspyii* and* Amarasca biguttula* are among the most dangerous piercing-sucking insects that cause damage to many crops, such as okra, sunflower, and cotton^18,19^; therefore, the need for controlling these pests using natural pesticides has become very important and has not been tried before using *E. paralias* fractions and isolated compounds. In this context, exploring the bioactive potential of *E. paralias* becomes highly significant, but there is a problem with the unclear, precise molecular mechanisms underlying its therapeutic effects. Specifically, the lack of integration between experimental and theoretical approaches has hindered a comprehensive understanding of structure–activity relationships, leaving a significant knowledge gap in the field. Therefore, the present study aims to systematically evaluate the antibacterial and insecticidal activities of extracts and isolated compounds from *E. paralias* using a combination of phytochemical analysis, quantum chemical assessments, and molecular docking studies. By integrating experimental and computational approaches, this study aimed to identify novel bioactive compounds with potential applications against microbial infections and pest infestations, thereby contributing to the advancement of safe and effective natural therapeutics.

## Experimental

### General

NMR analysis was performed using a Bruker spectrometer in deuterated pyridine, methanol, and DMSO at 500 megahertz for ^1^H and 100 megahertz for ^13^SPS:refid::bib13C NMR at the Faculty of Science, Mansoura University, and at 400 MHz for ^1^H and 100 MHz for ^13^C NMR at the Faculty of Pharmacy, Mansoura University. Gas chromatography-mass spectrometry (GC–MS) analysis was conducted using a GC-TSQ mass spectrometer (Thermo Scientific, Austin, TX, USA). Column chromatography was performed on Polyamide S6 (Merck), while preparative thin-layer chromatography (TLC) was carried out using silica gel GF254-coated plates (Merck) measuring 20 × 20 cm on aluminum sheets. Solvents; methanol, butanol, ethyl acetate, methylene chloride, and hexane were provided from ADVENT (CHEMBIO PVT.LTD.) company, India. Spray reagent; *P*-anisaldehyde-sulphuric acid reagent. Microbial strains were obtained from Mansoura University Center of Genetic Engineering and Biotechnology.

### Plant material

*E. paralias* (Ephorbiaceae) aerial parts were gathered in June 2021 from Marsa Matrouh, Cleopatra Bath, Egypt (31.373185 N-27.186673 E). According to Boulos (2000)^20^, Prof. Dr. Ibrahim Mashaly, Department of Botany, Faculty of Science at Mansoura University, identified the plant. A herbarium specimen was deposited in the Herbarium of Botany department, Faculty of Science, Mansoura University with the name of El-Rokh A. R samples.

### Extraction and isolation

Methanol was used to soak 7.5 kg of dried powdered *E. parlias* aerial parts, resulting in 1194.9 g of methanol extract. The extract was partitioned using separating funnel to afford four fractions; hexane (180.55 g), chloroform (34.67 g), ethyl acetate (31.79 g) and butanol (160.61 g). Seven compounds (**1**–**7**) were successfully isolated and identified. A portion of the hexane fraction was subjected to GC/MS analysis, resulting in the identification of thirty-four compounds (Table 1). Additionally, GC/MS analysis of the chloroform fraction led to the identification of thirteen compounds (Table 1).

### Characterization of isolated compounds (1–7)

#### *β*-sitosterol-3-*O-β*-d-glucoside (**1**)

A white powder, ^1^H NMR (500 MHz, C_5_D_5_N, *δ*_H_, ppm, *J*, Hz): *δ*_H_ 0.67 (s, 3H-18), 0.95 (s, 3H-19), 0.87 (d, *J* 7.0 Hz, 3H-26), 0.89 (d, *J* 6.8 Hz, 3H-27), 0.90 (t, *J* 7.3 Hz, 3H-29), 1.00 (d, *J* 6.4 Hz, 3H-21), 3.97 (m, H-3), 4.02 (m, H-5′), 4.10 (t, *J* 8.0 Hz, H-2′), 4.34 (m, H-3′), 4.31 (m, H-4′), 4.44 (dd, *J* 5.2, 11.8 Hz, H-6′), 4.60 (dd, *J* 2.1, 11.8 Hz, H-6′), 5.08 (d, *J* 8.0 Hz, H-1′), 5.35 (br s, H-6); ^13^C NMR (125 MHz, C_5_D_5_N) *δ*c 37.8 (C-1), 30.6 (C-2), 78.4 (C-3), 39.7 (C-4), 141.2 (C-5), 122.3 (C-6), 32.5 (C-7), 32.4 (C-8), 50.7 (C-9), 36.7 (C-10), 21.6 (C-11), 40.3 (C-12), 42.8 (C-13), 57.1 (C-14), 24.9 (C-15), 28.9 (C-16), 56.5 (C-17), 12.3 (C-18), 19.8 (C-19), 37.3 (C-20), 19.4 (C-21), 34.5 (C-22), 26.7 (C-23), 46.4 (C-24), 29.8 (C-25), 19.5 (C-26), 20.3 (C-27), 23.7 (C-28), 12.5 (C-29), 102.9 (C-1′), 75.7 (C-2′), 79.0 (C-3′), 72.0 (C-4′), 78.9 (C-5′), 63.2 (C-6′).

#### Genistein-4′-*β*-*O-*d-glucopyranoside (**2**)

Amorphous white powder, ^1^H NMR (500 MHz, CD_3_OD, *δ*_H_, ppm, *J*, Hz): *δ*_H_): *δ*_H_ 8.10 (s, H-2), 6.18 (br s, H-6), 6.27 (br s, H-8), 7.36 (d, *J* 7.1 Hz, 2H-2′, 6′), 6.86 (d, *J* 7.1 Hz, 2H-3′, 5′), 5.21 (d, *J* 6.4 Hz, H-1″), 3.25–3.85 (6H-2″-6″a,b).

#### Quercetin-3-*β*-*O-*d-glucoside (**3**)

^1^H NMR (500 MHz, CD_3_OD, *δ*_H_, ppm, *J*, Hz): *δ*_H_ 7.84 (d, *J* 1.5 Hz, H-2′), 7.58 (dd, *J* 1.5, 7.0 Hz, H-6′), 6.85 (d, *J* 7.0 Hz, H-5′), 6.37 (br s, H-8), 6.18 (br s, H-6), 5.12 (d, *J* 6.5 Hz, H-1″), 3.30–3.90 (6H-2″- 6″a,b).

#### Kaempferol-3-*O-α*-d-arabinopyranoside (**4**)

Yellow powder, ^1^H NMR (400 MHz, DMSO-*d*_6_, *δ*_H_, ppm, *J*, Hz): *δ*_H_ 5.74 (br s, H-6)**,** 5.91 (br s, H-8), 7.97 (d, *J* 8.5 Hz, 2H-2′, 6′), 6.84 (d, *J* 8.5 Hz, 2H-3′, 5′), 5.21 (d, *J* 5.2 Hz, H-1″), 3.19–3.72 (m, H2″- 5″).

#### Quercetin-3-*O-α*-d-arabinopyranoside (**5**)

Yellowish powder, ^1^H NMR (500 MHz, DMSO-*d*_6_, *δ*_H_, ppm, *J*, Hz): *δ*_H_ 6.09 (br s, H-6)**,** 6.29 (br s, H-8), 7.52 (s, H-2′), 6.82 (d, *J* 8.3, H-5′), 7.63 (d, *J* 8.3, H-6′)**,** 5.25 (d, *J* 5.0 Hz, H-1″), 3.77 (t, *J* 5.6 Hz, H-2″), 3,52 (m, H-3″), 3.65 (m, H-4″), 3.24 (d, *J* 10.2 Hz, H-5″*α*), 3.62 (m, H-5″*β*).

#### Genistein-8*-β*-C-glucoside (**6**)

Amorphous white powder, ^1^H NMR (500 MHz, DMSO-*d*_6_, *δ*_H_, ppm, *J*, Hz): *δ*_H_ 8.38 (s, H-2), 6.34 (s, H-6), 7.40 (d, *J* 7.8 Hz, 2H-2′, 6′), 6.85 (d, *J* 7.8 Hz, 2H-3′, 5′), 4.72 (d, *J* 8.8 Hz, H-1″), 4.02 (t, *J* 8.8 Hz, H-2″), 3.30 (t, *J* 8.8 Hz, H-3″), 3.26 (m, H-4″, 5″), 3.75 (d, *J* 11.2 Hz, H-6″a), 3.48 (m, H-6″b).

#### Hirsutissimiside B (**7**)

Colorless slice crystal, ^1^H NMR (500 MHz, CD_3_OD, *δ*_H_, ppm, *J*, Hz): *δ*_H_ 8.15 (s, H-2), 6.59 (d, *J* 1.0 Hz, H-6), 6.72 (d, *J* 1.0 Hz, H-8), 3.83 (s, OCH_3_), 7.30 (d, *J* 8.5 Hz, 2H-2′, 6′), 6.78 (d, *J* 8.5 Hz, 2H-3′, 5′), 5.07 (d, *J* 7.5 Hz, H-1″), 4.70 (d, *J* 2.2 Hz, H-1‴), 1.1 (d, *J* 6.1 Hz, 3H-6‴) and 3.15–3.75 (m, 6H-2″-6″)-*β*-d-glucose and 4H-2‴-5‴)-*α*-d-rhamnose).

### Antimicrobial activity (well diffusion method)

Five fractions and the isolated compounds were individually evaluated for antibacterial activity against a panel of Gram-positive (*Staphylococcus aureus, Staphylococcus epidermis, Bacillus subtilis*) and Gram-negative (*Escherichia coli, Klebsiella pneumonia*) bacteria. A solution with a concentration of 2.5 mg/ml was made independently by dissolving each fraction and the separated compounds in DMSO. The microbial inoculum is dispersed across the surface of the agar plate to inoculate it. Wells of 8 mm diameter were aseptically punched into the agar using a sterile cork borer, and 100 µL of each test solution was introduced into the wells. After a day of incubation at 36°C, the inhibitory zones in the Petri dishes were measured. Three duplicates of each treatment were made^21^. Gentamicin, dissolved in DMSO at a concentration 2.5 mg/mL, was used as a positive control following the same procedure**.**

The following formula was used to determine each treatment′s percentage activity index:

$${\text{\% Activity}}\;{\text{ Inde}} = { }\frac{{{\text{Zone }}\;{\text{of }}\;{\text{inhibition }}\;{\text{by}}\;{\text{test }}\;{\text{extract }}\;\left( {{\text{diametre}}} \right)}}{{{\text{Zone }}\;{\text{of }}\;{\text{inhibition }}\;{\text{by }}\;{\text{standard}}\;\left( {{\text{diametre}}} \right)}}\; \times \;{ }100$$

### Insecticidal activity (spray method)

Azadirachtin (Okios 3.2% EC) served as a positive control in evaluating the insecticidal activity of the methanol extract, its derived fractions, and isolated compounds against *Aphis gosspyii* and *Amrasca biguttula* using the spray approach on okra crops. Stock solutions were prepared using 0.1% Tween 80 in distilled water. Each bioassay included five concentrations of the test material, with four replicates per concentration. Ten aphids or jassids in each replicate were transferred to 4.0 cm-diameter okra leaves, which were then put in petri plates with 1.5% agar media. Two milliliters of each treatment were sprayed after 30 min. Death counts were recorded after 24 h^22^. The death percentages were corrected by Abbott (1925)^23^. The corrected mortality percentages of extract, fractions, and compounds were applied to probit analysis according to Finney^24^ to obtain the LC_50_, LC_90_ and their confidence limits, as well as the slope of the regression (LC-P) lines. Also, the efficiency of the extract, fractions, and separated compounds was evaluated by comparing the examined compounds or fractions with the most effective compound or fraction^25^ as follows:

$${\text{Toxicity}}\;{\text{ index}} = \frac{{{\text{LC}}50\;{\text{of }}\;{\text{the}}\;{\text{ most }}\;{\text{effective}}\;{\text{ compound}}\;{\text{ or }}\;{\text{extract}}}}{{{\text{LC}}50{ }\;{\text{of}}\;{\text{ the}}\;{\text{ tested }}\;{\text{compound }}\;{\text{or }}\;{\text{extract}}}} \times 100$$

### DFT modeling study

The studied nature derivatives **1–7** were geometrically optimized by applying B3LYP functional with the 6-31G(d) basis set methodology in Gaussian 09W program^26^ and their electronic and FMO′s have been examined through GaussView package^27^.

### Molecular docking study

Docking study as a supportive method for elucidating the interactions between a ligand and relevant active regions of a protein^28^. Investigations and calculations related to docking were performed utilizing the Molecular Operating Environment program MOE version 2019.0102. The docking setup included the following parameters: Placement method: Triangle Matcher, Initial scoring function: London dG, Refinement method: Rigid Receptor, Final scoring function: GBVI/WSA dG. Since EGFR is recognized as an epidermal growth factor receptor, the protein′s crystal structure (PDB ID: (1MOQ)) was acquired from the Protein Data Bank file (PDB). The crystal structure with PDB ID 1MOQ corresponds to Glucosamine-6-phosphate synthase (GlcN-6-P synthase) from *Escherichia coli*. This enzyme plays a crucial role in the biosynthesis of bacterial cell walls by catalyzing the conversion of fructose-6-phosphate to glucosamine-6-phosphate, a key step in the formation of peptidoglycan precursors. Due to its essential role in bacterial survival and absence in human metabolic pathways, GlcN-6-P synthase represents a promising antibacterial drug target. Thus, 1MOQ was selected as the target structure for molecular docking studies to evaluate the potential inhibitory activity of the compounds^28^. The docking process involves several steps: (1) elimination of heteroatoms and water from the target complex, (2) constructing the protein by incorporating missing hydrogens and automatically connecting and typing through optimizing the potential energy, and (3) executing the docking procedure. The docking was carried out within a grid box of 10 Å dimensions along the x, y, and z dimensions, centered on the ligand, utilizing MOE, with binding scores of the compounds ranked as **7** > **1** > **3** > **6** > **2** > **4** > **5**. Ultimately, the binding energies for ligand-receptor interactions were documented.

## Results and discussion

### Identification of compounds (1–7)

**Fig. 1:**
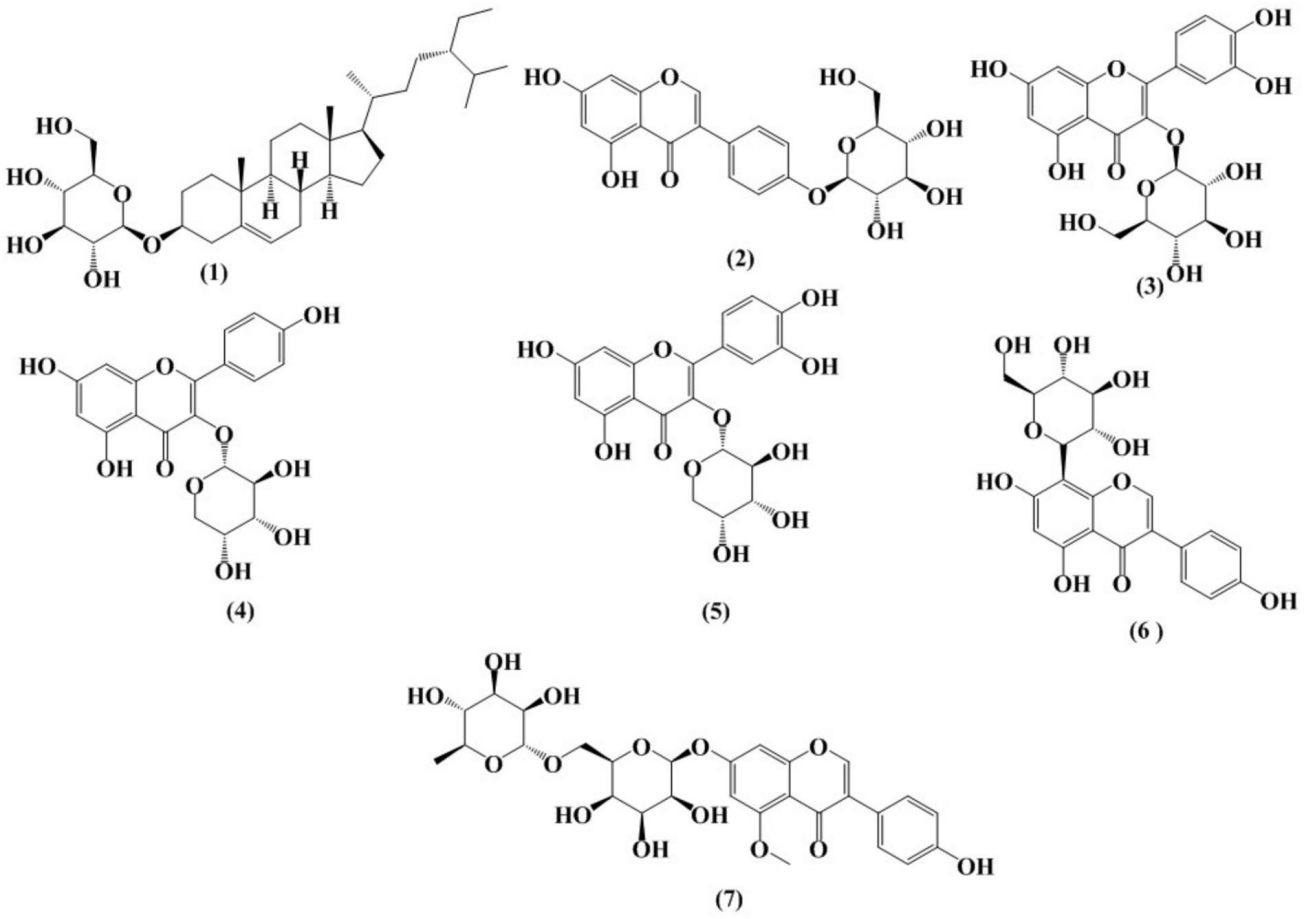
Chemical structures of compounds (**1**–**7**).

Compound **1** was isolated as a white powder and gave a reddish-violet color with a *p*-anisaldehyde spray reagent. ^1^H NMR spectrum showed six methyl signals, two singlets at *δ*_H_ 0.67 (s, 3H) and 0.95 (3H, s), three doublets at *δ*_H_ 1.00 (3H, d, *J* 6.4 Hz), 0.87 (3H, d, *J* 7.0 Hz) and 0.89 (3H, d, *J* 6.8 Hz), and one triplet at *δ*_H_ 0.90 (3H, t, *J* 7.3 Hz), indicating either steroid or triterpenoid. The H-3 signals at *δ*_H_ 3.97 (^1^H, m), and *δ*_c_ 78.4 that appeared as a multiplet in the proton nmr indicated a steroidal compound without the two methyl groups at C-4. An olefinic proton signal at *δ*_H_ 5.35 ppm and *δ*_C_ 122 ppm was found to be characteristic to H-6 of *β*-Sitosterol^29,30^. Also, the ^1^H and ^13^C NMR spectra (Supplementary Figs. S1, S1a, S1b S2, S2a, S2b) showed signals in the sugar regions and one anomeric H/C at *δ*_H_ 5.08 (^1^H, d, *J* 8.0 Hz), and *δ*_C_ 102.9 in agreement with a *β*-d-glucopyranoside^29^. By comparing the spectral data with those previously obtained from *Licania carii* leaves by Bilia et al. compound **1** was identified as *β*-sitosterol-3-*O-β*-d-glucoside^31–33^. Four compounds **2**, **3**, **4** and **5** (Fig. 1) were separated and identified by ^1^H NMR from ethyl acetate fraction. Compound **2** was isolated as an amorphous white powder that gave a yellow color with a para anisaldehyde. ^1^H NMR data (Supplementary Figs. S3 and S3a) showed the feature H-2 signal at *δ*_H_ 8.10 (s). Additionally, signals of H-6 and H-8 at *δ*_H_ 6.18 (br s) and 6.27 (br s), an AA′BB′ spin system of the ring B at *δ*_H_ 7.36 (*J* 7.1 Hz, H-2′,6′) and 6.86 (*J* 7.1 Hz, H-3′,5′) indicating glucosidation at 4′-OH. An anomeric proton signal appeared at *δ*_H_ 5.21 (^1^H, d, *J* 6.4 Hz, H-1″) indicating the glycosidic linkage was a beta linkage. Thus, it was identified by comparing with literature spectral data as genistein-4′-*O-β*-d-glucopyranoside^34^. ^1^H NMR spectrum of compound **3 (**Supplementary Figs. S3 and S3a) showed three aromatic signals for the ABX system at *δ*_H_ 7.84 (^1^H, d, *J* 1.5 Hz), 7.58 (^1^H, dd, *J* 1.5, 7.0 Hz), and 6.85 (^1^H, d, *J* 7.0 Hz) indicating the presence of 1,2,4-trisubstituted benzene ring. Also, the presence of two signals at *δ*_H_ 6.18 (^1^H, s) and 6.37 (^1^H, s) indicates the presence of quercetin skeleton and the glycosidic linkage of the anomeric proton at *δ*_H_ 5.12 (^1^H, d, *J* 6.5 Hz, H-1″) was beta linkage at C-3. The above data agreed with quercetin-3-*O-β*-glucoside that was isolated previously from *Curatella Americana*^35^. Compound **4** was isolated as a yellow powder that gave dark yellow color with the *p*-anisaldehyde spray reagent. ^1^H NMR spectrum of compound **4** (Supplementary Figs. S4 and S4a) showed aromatic signals for the AA″BB′ system at [*δ*_H_ 6.84 and 7.97 (each, 2H, d, *J* 8.5 Hz)]. Also, signals at *δ*_H_ 5.74 (^1^H, br s) and 5.91 (^1^H, br s) indicating the presence of kaempferol aglycon with an anomeric proton signal appeared at *δ*_H_ 5.21 (^1^H, d, *J* 5.2 Hz, H-1″), thus, it was identified as kaempferol-3-*O-α*-d-arabinopyranoside^36–38^ (Fig. 1). Compound **5** was isolated as a yellow powder that gave dark yellow color with the *p*-anisaldehyde spray reagent. ^1^H NMR spectrum of compound **5** (Supplementary Figs. S5 and S5a) showed a signal pattern similar to that of the kaempferol glycoside** 4** except for the presence of ABX spin system instead of AA′BB′ system at [*δ*_H_ 7.52 (^1^H, s), 6.82 (^1^H, d, *J* 8.3 Hz), and 7.63 (^1^H, d, *J* 8.3 Hz)], which agree with quercetin-3-*O-α*-d-arabinopyranoside^39–41^. Compound **6** was isolated as a yellowish powder that gave dark yellow color with the *p*-anisaldehyde spray reagent. It was proved by ^1^H NMR analysis (Supplementary Figs. S6, S6a,) that showed a singlet at *δ*_H_ 8.38 ppm characteristic of isoflavone. The shikimate ring was represented by AA′BB′ system signals at *δ*_H_ 6.85 (2H, d, *J* 7.8 Hz) and 7.40 (2H, d, *J* 7.8 Hz), while the acetate ring was represented by a singlet at *δ*_H_ 6.34 ppm of H-6. An anomeric proton signal appeared at *δ*_H_ 4.72 (^1^H, d, *J* 8.8 Hz) indicating the presence of C-*β*-d-glucopyranose at C-8^42^. APT ^13^C NMR and HSQC spectra (Supplementary Figs. S7 and S8) showed the expected 21 signals of carbon. Thus, it was identified and confirmed as genistein-8-C-glucoside (Fig. 1) by comparing its spectral data with those of previously isolated from *Pueraria lobata*^42,43^. Compound **7** was isolated as colorless slice crystal that gave dark yellow color with the *p*-anisaldehyde spray reagent. ^1^H NMR spectrum of compound **7** (Supplementary Figs. S9 and S9a) showed signals for a proton at *δ*_H_ 8.15 (1H, s) and for AA′BB′ spin system at [*δ*_H_ 6.78 and 7.30 (each, 2H, d, *J* 8.5 Hz)] representing the shikimate ring, which corresponds to an isoflavone skeleton. The acetate ring was represented by a pair of down-field shift signals at *δ*_H_ 6.59 (1H, d, *J* 1.0 Hz) and *δ*_H_ 6.72 (1H, d, *J* 1.0 Hz) indicating the 5,7-dioxygenation^44^. The two substituents were concluded from the proton signal at *δ*_H_ 3.83 (3H, s, OCH_3_) corresponding to a methoxyl group, especially after the disappearance of 5-OH signal in the spectrum, and two anomeric proton signals of sugars at *δ*_H_ 5.03 (1H, d, *J* 7.5 Hz, H-1″), and 4.70 (1H, d, *J* 2.2 Hz, H-1‴) with two glycosidic linkage, one of them was beta configuration, and the other was an alpha configuration. Thus, it was identified by comparing with literature spectral data as hirsutissimiside B^44^ (Fig. 1).

### GC/MS analysis

Hexane and chloroform fractions were analysed by the GC/MS technique that resulted in identification of 34 and 13 compounds, respectively (Table 1). The MS spectra of compounds were compared with their analogous compounds contained in NIST main library and the Wiley Registry mass spectral library. The identified compounds can be classified into one α-tocopheroid (**44)**, sixteen acetogenins **(9, 13, 19**, **23**, **24, 28**, **29, 30**, **31**, **32, 33**, **38, 39**, **41**, **42**, and** 47)**, two triterpenes** (48** and** 49)**, three sesquiterpenes **(14**, **15** and **17)**, four monoterpenes **(8**, **10**, **18** and **22)**, four sterols **(35**, **43**, **45** and **46)**, five diterpenes **(20**, **25**, **27**, **34** and **37)**, seven phenolic compounds **(11**, **12**,** 16**, **21**, **26**, **36** and **40)**. The hexane fraction showed greater compounds diversity and was predominantly composed of volatile and less polar molecules, such as 17-pentatriacontene (7.87%), eugenol (7.52%), barrigenol (6.28%), and ursane-3,12-diol (4.55%). In comparison, the chloroform fraction had fewer but highly enriched bioactive oxygenated compounds that were little high polar than compounds in hexane fraction and with major compounds such as eugenol (25.8%)**,** barrigenol (14.48%)**,** (E)-coniferyl alcohol (11.2%), and trans-sinapyl alcohol (8.04%).

**Table 1:** Chemical constituents identified by GC/MS technique from hexane and chloroform fractions of *Euphorbia paralias* L.

Compound name	R_t_	Mol. Formulae	Mol. wt	% of compounds in the fractions		Reported bioactivity	References
				Hexane	CHCl_3_		
Cuminaldehyde (**8**)	10.64	C_10_H_12_O	148	0.37	–	Antimicrobial, antioxidant, insecticidal	^45^
Trans-2-decenal (**9**)	11.17	C_10_H_18_O	154	0.80	–	Antimicrobial, insecticidal, anticancer	^46^
Hexahydro-2,5-methano-1H-inden-7(4H)-one (**10**)	12.02	C_10_H_14_O	150	0.27	–	Antimicrobial, antioxidant	^47^
Eugenol (**11**)	13.52	C_10_H_12_O_2_	164	7.52	25.8	Antimicrobial, antioxidant, anti-inflammatory, insecticidal	^45,48^
Isoeugenol (**12**)	15.93	C_10_H_12_O_2_	164	–	2.98	Antimicrobial, antioxidant, anticancer, insecticidal	^48^
Trans-2-tetradecenal (**13**)	18.73	C_14_H_26_O	210	1.48	–	Insecticidal	^46,49^
*β*-caryophyllene oxide (**14**)	18.96	C_15_H_24_O	220	3.42	–	Antimicrobial, insecticidal, anticancer	^50^
caryophylla-4(12),8(13)-dien-5α-ol (**15**)	20.27	C_15_H_24_O	220	0.52	–	–	–
O-paradol (**16**)	20.43	C_11_H_14_O_3_	194	–	4.77	Antimicrobial, antioxidant, anti-inflammatory, anticancer	^51^
Ar-tumerone (**17**)	20.84	C_15_H_20_O	216	0.90	–	Antimicrobial, anti-inflammatory, anticancer	^50^
*α,β*-Dihydro-*β*-ionone (**18**)	21.71	C_13_H_22_O	194	–	3.04	–	–
13,16-Octadecadiynoic acid, methyl ester (**19**)	22.19	C_19_H_36_O_2_	290	0.48	–	–	–
Retinal (**20**)	22.28	C_20_H_28_O	284	0.26	–	–	–
(E)-coniferyl alcohol (**21**)	22.64	C_10_H_12_O_3_	180	–	11.2	–	–
(−)-Loliolide (**22**)	23.22	C_11_H_16_O_3_	196	–	3.64	Antioxidant, anti-inflammatory	^52^
Palmitic acid, methyl ester (**23**)	26.34	C_17_H_34_O_2_	270	4.18	–	–	
Linoleic acid (**24**)	27.42	C_18_H_32_O_2_	280	0.72	–	Antimicrobial	^53^
Manoyl oxide (**25**)	27.58	C_20_H_34_O	290	2.68	–	Antimicrobial, antiprotozoal, anti–leishmanial, anti-inflammatory	^54^
Trans-sinapyl alcohol (**26**)	27.73	C_11_H_14_O_4_	210	–	8.04	Antimicrobial, antifungal, antioxidant, **anti-inflammatory, anti-nociceptive**	^55^
Colensenone (**27**)	27.80	C_19_H_30_O_2_	290	1.98	5.3	–	–
8,11-octadecadienoic acid, methyl ester (**28**)	29.49	C_19_H_34_O_2_	294	2.21	–	Antimicrobial	^53^
Oleic acid, methyl ester (**29**)	29.64	C_19_H_36_O_2_	296	4.10	–	Antimicrobial, Antioxidant, anti-inflammatory, Anticancer activities	^53^
1-Monopalmitin (**30**)	29.84	C_19_H_38_O_4_	330	0.22	–	Antimicrobial, Anticancer activities	^56^
9-Methyl-heptadecanoic acid, methyl ester (**31**)	30.14	C_19_H_38_O_2_	298	0.83	–	–	–
Oleic acid (**32**)	30.66	C_18_H_34_O_2_	282	0.43	–	Antimicrobial, anti-inflammatory, anticancer	^57^
Linoleic acid, ethyl ester (**33**)	31.49	C_20_H_36_O_2_	308	0.43	–	Anti-inflammatory, anti–Melanogenesis, skin barrier improvement	^53^
2-Oxomanoyl oxide (**34**)	31.63	C_20_H_32_O_2_	304	1.84	–	Antimicrobial, vasorelaxant Activity	^54^
Pregnane-3,17,20-triol (**35**)	32.25	C_21_H_36_O_3_	336	1.72	–	–	–
Shogaol (**36**)	32.96	C_17_H_24_O_3_	276	1.00	6.61	Antimicrobial, antioxidant, anti-inflammatory, anticancer	^51^
8A,13-Epoxy-2-oxolabdan-20-al (**37**)	33.34	C_20_H_32_O_3_	320	1.21	–	antimicrobial, anti-inflammatory, anticancer	^54^
Henicosanoic acid (**38**)	33.64	C_21_H_42_O_2_	326	1.85	–	–	–
2-Mono-linolein (**39**)	34.02	C_21_H_38_O_4_	354	0.21	–	Antimicrobial, antiviral	^58^
Gingerol (**40**)	34.56	C_17_H_26_O_4_	294	–	4.94	Antimicrobial, antioxidant, anti-inflammatory, anticancer activities, cardiovascular Health	^51^
1,2-Dipalmitin (**41**)	36.62	C_35_H_68_O_5_	568	1.56	–	–	–
2-mono-olein (**42**)	39.42	C_21_H_40_O_4_	356	3.56	–	Antibacterial, antioxidant and cytoprotective	^59^
γ-Sitosterol (**43**)	40.38	C_29_H_50_O	414	0.56	4.64	Antibacterial, antioxidant, anti-inflammatory, anticancer, cholesterol lowering	^60^
α-Tocospiro A (**44**)	40.88	C_29_H_50_O	462	1.69	–	Antioxidant, anti-inflammatory, anticancer, anti–diabetic	^61^
Ursodeoxycholic acid (**45**)	40.95	C_24_H_40_O_4_	392	–	4.52	Hepatoprotective and cholagogue, lipid lowering	^62^
Cholestane-3,6,7-triol (**46**)	41.08	C_27_H_48_O_3_	420	2.73	–	Biomarker for Niemann–Pick Type C Disease, Pro–oxidant and cytotoxic Properties	^63^
17-Pentatriacontene (**47**)	41.54	C_35_H_70_	490	7.87	–	Antibacterial, antioxidant, anti-inflammatory	^59^
Ursane-3,12-diol (**48**)	41.78	C_30_H_52_O_2_	444	4.55	–	Antibacterial, antioxidant, anti-inflammatory, cytotoxic/antitumor	^64^
Barrigenol (**49**)	42.59	C_30_H_50_O_6_	506	6.28	14.48	Anti–tumor/cytotoxic activity, neuroprotective, anti-inflammatory	^64^

### Electron density distribution analysis (DFT) for compounds 1–7

**Fig. 2:**
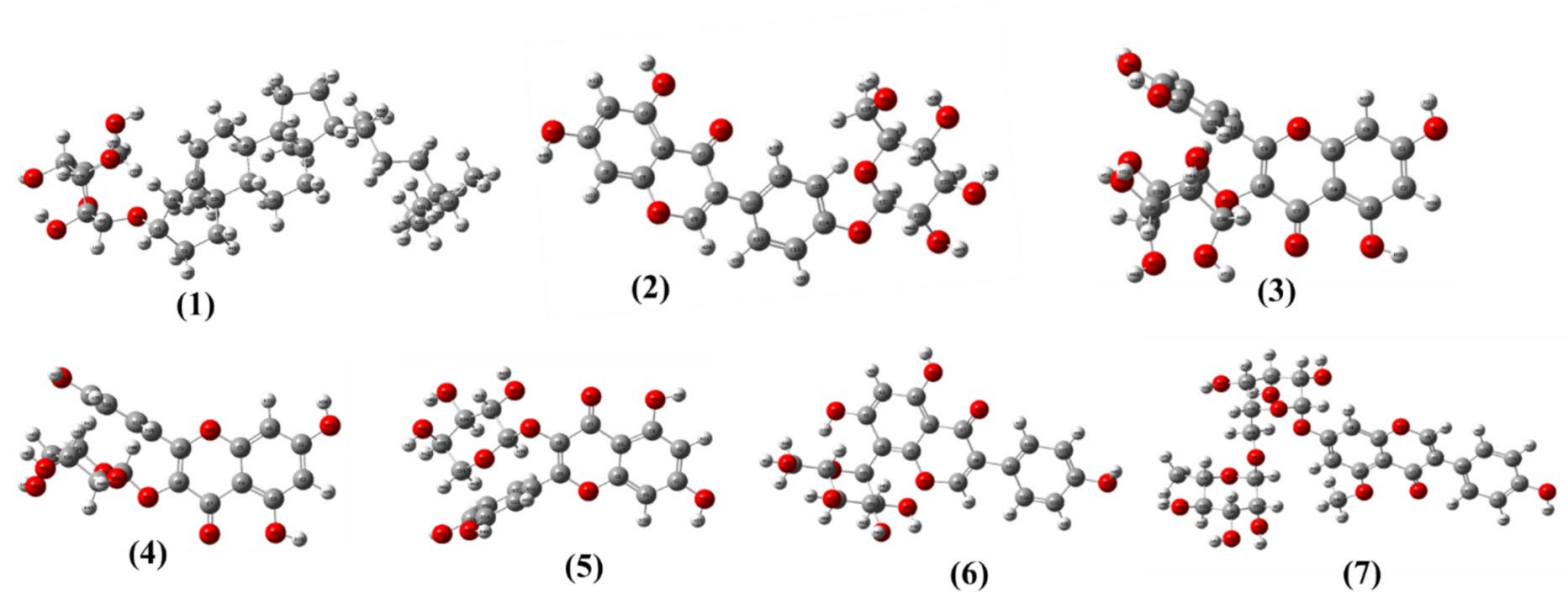
Optimized geometry structures of compounds **1–7**.

The optimized molecular structures of compounds **1–7** (Fig. 2) were determined using Density Functional Theory (DFT) calculations, ensuring that each molecule attained its lowest energy conformation. The structural optimization was performed using the B3LYP functional with the 6-31G(d) basis set^26^, leading to stable configurations with minimized steric hindrance and optimized bond angles. The optimized structures reveal key molecular features that influence biological interactions, such as planarity, conjugation, and hydrogen bonding potential. Compounds containing flavonoid skeletons, such as** 2**, **3**, **4**, and **5** displayed nearly planar conformations, enhancing their π–π stacking interactions with biological targets. These structural attributes facilitate strong binding with bacterial enzymes and receptor sites, which contribute to their antimicrobial and insecticidal activities. Compound **1** adopted a relatively rigid, non-planar conformation, influencing its ability to integrate into microbial membranes and disrupt their integrity. The optimized structures also reveal the spatial distribution of functional groups, which play a crucial role in molecular interactions^27^. For instance, compounds **6** and **7** exhibited well-defined hydrogen bond donors and acceptors, which enhance their ability to inhibit bacterial enzymes through strong electrostatic interactions. The presence of glycosidic linkages in these compounds contributes to their solubility and bioavailability, further impacting their biological efficacy. The optimized molecular geometries of compounds **1–7** highlight structural features that govern the biological activity of these compounds, reinforcing their potential as antimicrobial and insecticidal agents.

**Fig. 3:**
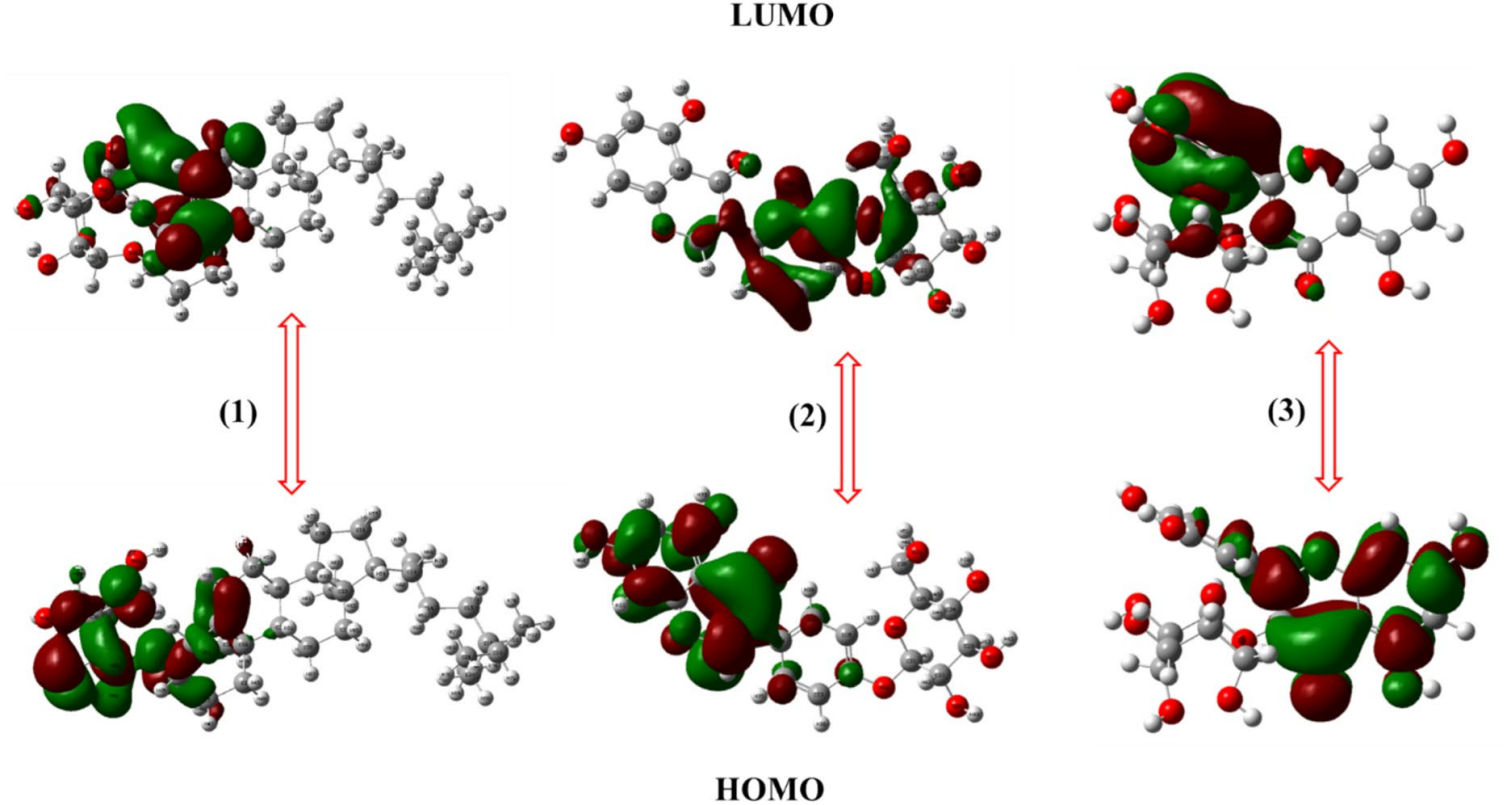
FMO levels for compounds **1–3.**

**Fig. 4:**
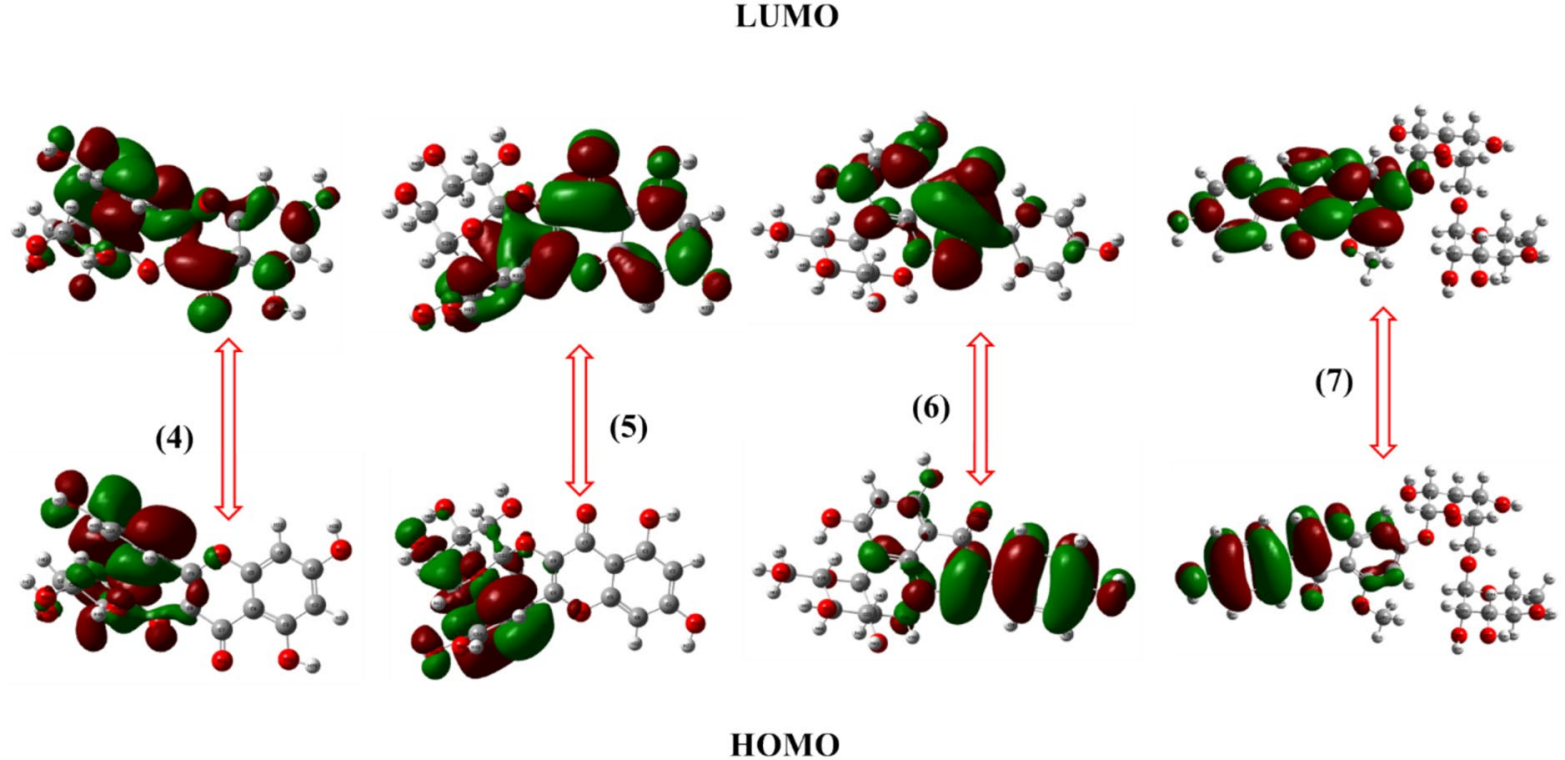
FMO levels for of compounds **4–7.**

The Frontier Molecular Orbital (FMO) analysis provides insights into the electronic properties of the isolated compounds from *Euphorbia paralias* (Figs. 3, 4), particularly their Highest Occupied Molecular Orbital (HOMO) and Lowest Unoccupied Molecular Orbital (LUMO) distributions^65^. The HOMO energy level represents the molecule′s ability to donate electrons, while the LUMO energy level indicates its ability to accept electrons, with the energy gap (ΔE = E_LUMO_ − E_HOMO_) serving as a key determinant of molecular reactivity and stability^65^. A small HOMO–LUMO gap suggests high reactivity and a greater likelihood of interactions with biological macromolecules, whereas a larger gap implies chemical stability but potentially lower bioactivity. The seven isolated compounds exhibited distinct HOMO and LUMO energy levels, influencing their ability to interact with microbial cell walls, proteins, and insects nervous system. Compound** 1** displayed a moderate HOMO–LUMO distribution, indicating balanced reactivity and strong interactions with bacterial membranes. Compounds **2** and **3** had higher HOMO energy levels, enhancing their electron donor capability, which contributes to strong bacterial protein interactions and insecticidal activity.

Compounds** 4** and **5** exhibited moderate HOMO–LUMO gaps, promoting their binding affinity to microbial proteins, leading to potent antibacterial effects. Compounds **6** and **7** demonstrated low LUMO energy levels, suggesting strong electrophilic properties that facilitate enzyme inhibition in bacterial and insecticidal systems. The electron distribution in these compounds highlights their potential interactions with biological targets, reinforcing their antimicrobial and insecticidal capabilities, Thus, the FMO analysis substantiates the observed biological activities, confirming that the electronic configurations of these compounds play a crucial role in their reactivity and effectiveness^65^.

### Molecular electrostatic potential (MEP) of compounds 1–7

**Fig. 5:**
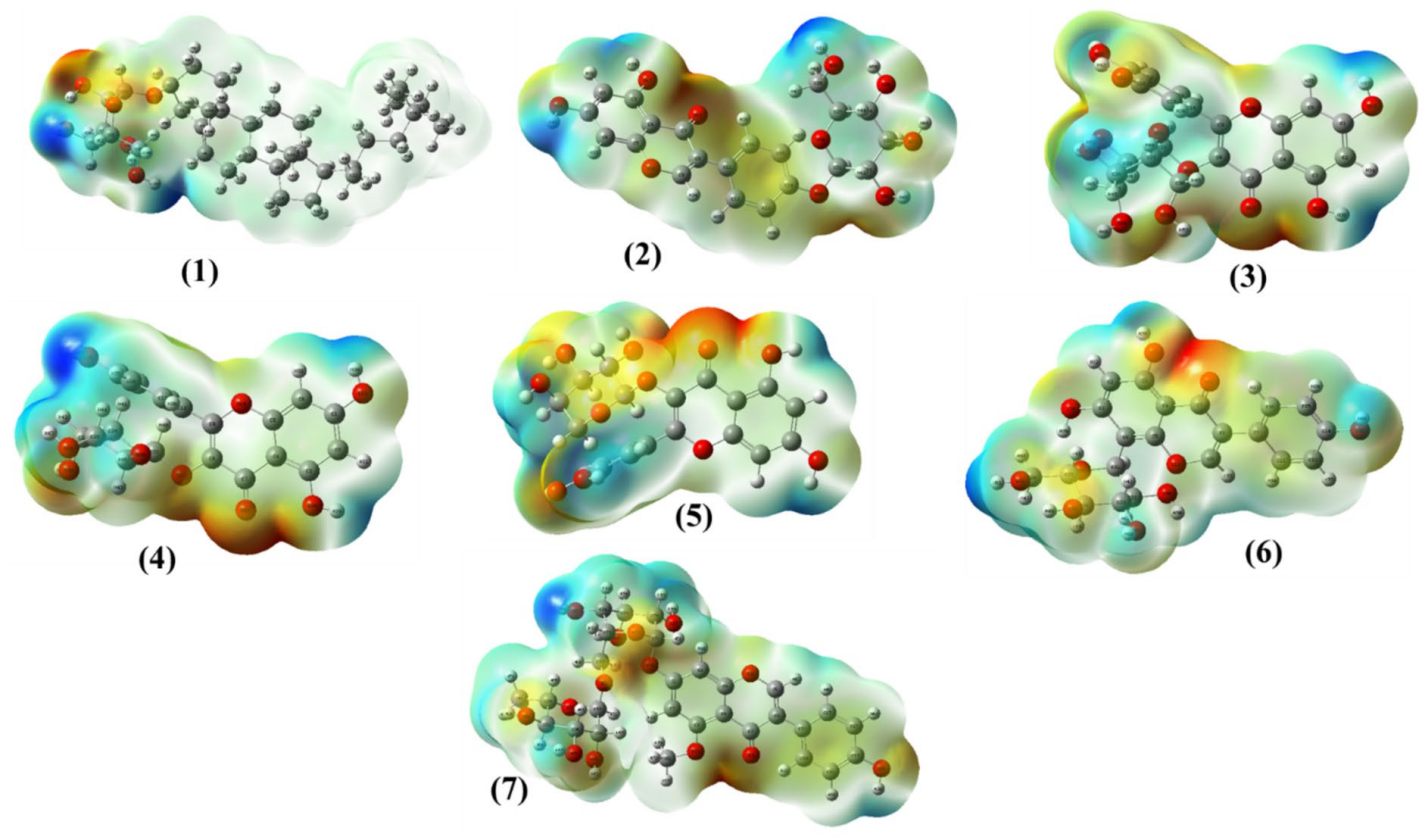
MEP simulations of compounds **1–7.**

To better understand the electronic features underlying the observed biological activity, we performed (MEP) analysis on compounds **1–7** (Fig. 5). MEP maps provide a spatial representation of the charge distribution across the molecular surface and are widely employed to predict sites of electrophilic and nucleophilic reactivit^66^. These maps also offer valuable insight into how compounds may engage in non-covalent interactions with biological macromolecules such as enzymes and microbial membranes. The MEP surfaces revealed distinct charge distributions that qualitatively correlate with variations in biological activity across the compound series. Compound **1** exhibited a relatively neutral electrostatic profile, suggesting limited potential for strong polar interactions. By contrast, compounds **2** and **3** showed marked regions of negative potential centered around oxygen-rich moieties, including hydroxyl and carbonyl groups, which may support hydrogen bonding interactions with enzyme residues. Compounds **4** and **5** featured even more pronounced electron-rich zones, which are typically associated with functional groups capable of acting as hydrogen bond acceptors. These features are often critical for electrostatic interactions with nucleophilic or positively charged residues within microbial targets. Compounds **6** and **7** displayed both electron-rich and electron-deficient regions, reflecting a more balanced electrostatic profile. This dual distribution may facilitate diverse binding interactions and could explain their stronger docking affinities and biological performance in vitro. Electron-rich regions on the MEP surface, commonly associated with electronegative atoms such as oxygen and nitrogen, play a fundamental role in the molecular recognition process. Functional groups such as carbonyl (C=O), hydroxyl (–OH), and amino (–NH₂) appear as localized areas of negative potential and often serve as hydrogen bond acceptors. These sites are well positioned to engage in electrostatic interactions with electron-deficient residues such as serine, lysine, or catalytic metal cofactors-within protein active sites. Such interactions can stabilize ligand-target complexes and may contribute to inhibition of enzyme activity. For instance, a carbonyl oxygen can form strong, directional hydrogen bonds that are critical for maintaining binding orientation and strength. Accordingly, compounds exhibiting accessible and well-defined electron-rich surfaces are likely to display enhanced interaction potential with biological targets.

Collectively, these results suggest that charge distribution as visualized through MEP maps may influence the observed in vitro antimicrobial behavior by modulating the ability of these molecules to form stabilizing interactions with enzymatic and cellular targets. While these findings offer meaningful insight into potential mechanisms of action, they are based on computational and in vitro analyses. As such, further in vivo studies and mechanistic validation are necessary to substantiate the functional relevance of these interactions and to fully assess the therapeutic potential of these compounds.

### Chemical reactivity parameters′ study of compounds 1–7

The quantum chemical parameters of compounds **1–7**, computed using DFT at the B3LYP/6-31G(d) level, provide valuable insights into their electronic structure, stability, and biological activity potential^65^. The HOMO–LUMO energy gap (ΔE) is a fundamental descriptor of chemical reactivity, influencing molecular stability and charge transfer capability. A smaller HOMO–LUMO gap facilitates easy electronic excitation, making a compound more chemically reactive as shown in Table 2. Among the compounds studied, compound **7** exhibits the smallest HOMO–LUMO gap (ΔE = 1.643 eV), confirming that it is the most reactive and least stable. This increased reactivity may enhance its biological interactions, particularly in enzyme inhibition and microbial cell wall penetration. Conversely, compound **6** has the largest energy gap (ΔE = 2.270), indicating higher stability and lower reactivity, which suggests a lesser biological impact. Chemical hardness (η) and chemical softness (σ) describe the resistance to electron exchange and tendency to react. Harder molecules (higher η) resist charge transfer, while softer molecules (higher σ) readily participate in interactions. The hardness values follow the order compound **6** (1.135 eV)** > **compound** 3** (1.134 eV)** > **compound** 4** (1.125 eV)** > **compound** 1** (1.074 eV)** > **compound** 7** (0.821 eV), confirming that compound **7** is the softest and most chemically reactive. This suggests a strong affinity for biological interactions, as softer molecules easily adapt to enzymatic and receptor environments, improving antimicrobial and insecticidal efficiency^67^. Dipole moment (D) plays a crucial role in determining polarity, solubility, and interaction strength. A higher dipole moment enhances bioavailability and molecular affinity for biological targets. Among the studied compounds, compound **7** has the highest dipole moment (7.562 D), indicating strong intermolecular interactions and high solubility. In contrast, compound** 6** has the lowest dipole moment (6.907 D), suggesting reduced solubility and lower bioactivity. Since higher dipole moments improve molecular dispersion in biological environments, compound **7** is expected to exhibit superior biological performance. Electronegativity measures the ability to attract electrons, while chemical potential reflects electron donation capacity**.** The electronegativity values follow the order ranged from (3.564–4.345 eV), confirming that compound **7**, with the lowest electronegativity, has the highest electron-donating ability. Electron donation plays a key role in charge transfer interactions, including enzyme inhibition and redox reactions involved in antimicrobial activity. The chemical potential (μ = − 3.564 eV for compound** 7**) further supports its higher biological activity, as compounds with higher chemical potential exhibit stronger interactions with biological targets. The correlation between quantum chemical descriptors and biological activity strongly suggests that higher chemical reactivity, lower hardness, higher dipole moment, and lower electronegativity contribute to enhanced antimicrobial and insecticidal properties. Among the studied compounds, compound **7** emerges as the most biologically promising, as its quantum descriptors strongly support enhanced solubility, high reactivity, and superior interaction with biological targets. In contrast, compound **6**, with the highest HOMO–LUMO gap, greatest hardness, and lowest dipole moment, is predicted to have the weakest biological activity. This study highlights the importance of quantum parameters in predicting biological activity, demonstrating that DFT calculations can serve as a reliable tool in drug discovery and bioactive compound optimization. The results suggest that compound **7** is the most suitable candidate for further biological evaluation, as its high reactivity, enhanced solubility, and strong electronic properties position it as a potent antimicrobial and insecticidal agent. DFT offers valuable insights into the electronic structure, stability, and reactivity of the molecules. Key descriptors such as HOMO–LUMO energy gap, dipole moment, and (MEP) maps help identify electrophilic and nucleophilic regions, which may correspond to interaction sites observed in docking.

**Table 2:** Quantum parameters for compounds** 1–7** by the B3LYP/6-31G(d) method.

Quantum parameter	Compounds						
	7	6	5	4	3	2	1
E(HOMO)/eV	− 4.386	− 5.480	− 5.050	− 5.117	− 5.143	− 4.970	− 4.967
E(LUMO)/eV	− 2.743	− 3.210	− 3.192	− 2.867	− 2.875	− 2.856	− 2.819
Energy/eV	1.643	2.270	1.858	2.250	2.268	2.114	2.148
Ionization energy, IP (eV)	4.386	5.480	5.050	5.117	5.143	4.970	4.967
Electron affinity, EA (eV)	2.743	3.210	3.192	2.867	2.875	2.856	2.819
Electronegativity, *χ* (eV)	3.564	4.345	4.121	3.992	4.009	3.913	3.89
Chemical potential (μ)	− 3.564	− 4.345	− 4.121	− 3.992	− 4.009	− 3.913	− 3.89
Chemical Softness (s)/eV	1.218	0.881	1.076	0.888	0.567	0.946	0.931
Chemical hardness (*ƞ*)/eV	0.821	1.135	0.929	1.125	1.134	1.057	1.074
Dipole moment/Debye	7.562	6.907	7.467	7.160	7.089	7.359	7.207

Where, IP =  −E_HOMO_, EA = −E*μ* =  −*χ* = (*E*_*LUMO*_ + *E*_*HOMO*_/2), *η* = (*E*_*LUMO*_ − *E*_*HOMO*_/2), s = 1/2* η*.

### Molecular docking study for compounds 1–7

Docking trials for all the scaffolds were carried out to comprehend the potential contact of the phytochemical ligands **1–7** on the GlcN-6-P synthase (ID: 1MOQ)^28^. The docked complexes with the highest binding affinities are shown in Table 3, which also presented the docking scores, bond distances, and interactions of the ligands with certain amino acids. The tabulated results indicated that the compounds under investigation exhibited optimal fitting within the protein’s active region, with binding energy scores ranging from − 7.0972 to − 8.6119 kcal/mol. Furthermore, the native ligand **GLP** (Glucosamine-6-phosphate, GLP) co-crystallized with the target protein (1MOQ) was used as the reference ligand for docking validation. This ligand served as a control to assess the reliability of the docking protocol through re-docking studies.

**Table 3:** Predictive docking scores and particular interactions of the ligands and the target protein.

Cpd. no	Binding energy (S) Kcal/mol	RMSD	Distance (Å)	Binding interactions		
				Ligand	Receptor	Interaction type
1	− 7.9924	1.9525	2.95	**O20**-**atom of OH group**	**Ser 303**	**H**-**donor**
			3.29	**O20**-**atom of OH group**	**Gln 348**	**H**-**acceptor**
2	− 7.5991	1.1785	2.91	**O18**-**atom of OH group**	**Glu 608**	**H**-**donor**
			2.95	**O27**-**atom of OH group**	**Glu 488**	**H**-**donor**
			2.92	**O29**-**atom of OH group**	**Glu 488**	**H**-**donor**
			2.79	**O30**-**atom of OH group**	**Val 399**	**H**-**donor**
			3.02	**O31**-**atom of OH group**	**Lys 603**	**H**-**donor**
			2.99	**O27**-**atom of OH group**	**Ser 401**	**H**-**acceptor**
			3.50	**O30**-**atom of OH group**	**Lys 603**	**H**-**acceptor**
			3.74	**Benzene ring**	**Cys 300**	**π**-**H**
3	− 7.9167	1.1720	2.79	**O20**-**atom of OH group**	**Thr 302**	**H**-**donor**
			2.81	**O29**-**atom of OH group**	**Ser 303**	**H**-**donor**
			2.81	**O31**-**atom of OH group**	**Cys 300**	**H**-**donor**
			3.25	**O13**-**atom of carbonyl group**	**Val 605**	**H**-**acceptor**
			3.11	**O29**-**atom of OH group**	**Ser 303**	**H**-**acceptor**
4	− 7.2895	1.8506	2.85	**O18**-**atom of OH group**	**Val 399**	**H**-**donor**
			3.20	**O20**-**atom of OH group**	**Asp 354**	**H**-**donor**
			2.71	**O28**-**atom of OH group**	**Cys 300**	**H**-**donor**
			2.92	**O29**-**atom of OH group**	**Ser 303**	**H**-**donor**
			2.98	**O30**-**atom of OH group**	**Thr 352**	**H**-**donor**
			3.05	**O18**-**atom of OH group**	**Ser 401**	**H**-**acceptor**
			3.32	**O30**-**atom of OH group**	**Ser 349**	**H**-**acceptor**
			2.98	**O30**-**atom of OH group**	**Ser 349**	**H**-**acceptor**
5	− 7.0972	1.8358	2.77	**O18**-**atom of OH group**	**Ser 347**	**H**-**donor**
			2.84	**O29**-**atom of OH group**	**Val 399**	**H**-**donor**
			2.73	**O30**-**atom of OH group**	**Val 399**	**H**-**donor**
			2.65	**O31**-**atom of OH group**	**Lys 603**	**H**-**donor**
			2.84	**O28**-**atom of OH group**	**Ser 349**	**H**-**acceptor**
			3.11	**O28**-**atom of OH group**	**Thr 302**	**H**-**acceptor**
			2.88	**O29**-**atom of OH group**	**Ser 401**	**H**-**acceptor**
6	− 7.7457	1.4663	2.84	**O18**-**atom of OH group**	**Glu 488**	**H**-**donor**
			2.95	**O20**-**atom of OH group**	**Asp 354**	**H**-**donor**
			3.39	**O27**-**atom of OH group**	**Gln 348**	**H**-**acceptor**
			3.21	**O29**-**atom of OH group**	**Ser 349**	**H**-**acceptor**
			4.44	**Pyrone ring**	**Gly 301**	**π**-**H**
7	− 8.6119	1.7515	3.25	**O38**-**atom of OH group**	**Glu 488**	**H**-**donor**
			2.72	**O39**-**atom of OH group**	**Val 399**	**H**-**donor**
			3.29	**O37**-**atom of OH group**	**Thr 302**	**H**-**acceptor**
			3.06	**O38**-**atom of OH group**	**Ser 401**	**H**-**acceptor**
			3.01	**O39**-**atom of OH group**	**Lys 603**	**H**-**acceptor**
			3.94	**Benzene ring**	**Lys 487**	**π**-**H**
GLP	− 6.1426	1.3872	2.84	**N16**-** NH**_**2**_** group**	**Glu 488**	**H**-**donor**
			2.79	**O29**-**atom of OH group**	**Ser 303**	**H**-**donor**
			3.39	**O14**-**atom of OH group**	**Gly 301**	**H**-**acceptor**
			2.88	**O14**-**atom of OH group**	**Thr 302**	**H**-**acceptor**
			3.49	**N16**-**NH**_**2**_** group**	**Ala 400**	**H**-**acceptor**
			3.01	**N16**-**NH**_**2**_** group**	**Ser 401**	**H**-**acceptor**
			3.22	**O19**-**atom of OH group**	**Lys 603**	**H**-**acceptor**
			3.48	**O26**-**atom of P=O group**	**Ser 347**	**H-acceptor**
			3.17	**O26**-**atom of P=O group**	**Ser 347**	**H**-**acceptor**
			3.17	**O26**-**atom of P=O group**	**Thr 352**	**H**-**acceptor**
			3.18	**O27**-**atom of OH group**	**Ser 349**	**H**-**acceptor**

**Fig. 6:**
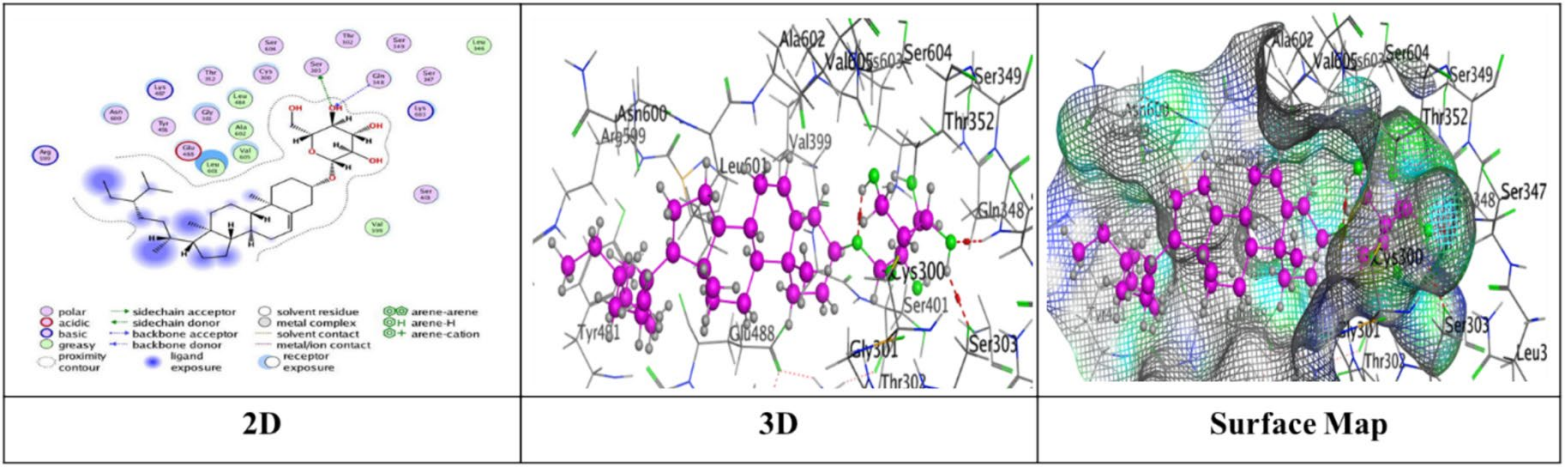
The interactions of compound **1** with active sites of (PDB ID: 1MOQ).

Compound **1** exhibited a significant binding affinity (S = in which O20 wherein the O20-atom of the hydroxyl group established two hydrogen bonds − 7.9924 kcal/mol), one H donor bond with Ser 303, and one H-acceptor bond with Gln 348 through RMSD value = 1.9525 (Fig. 6).

**Fig. 7:**
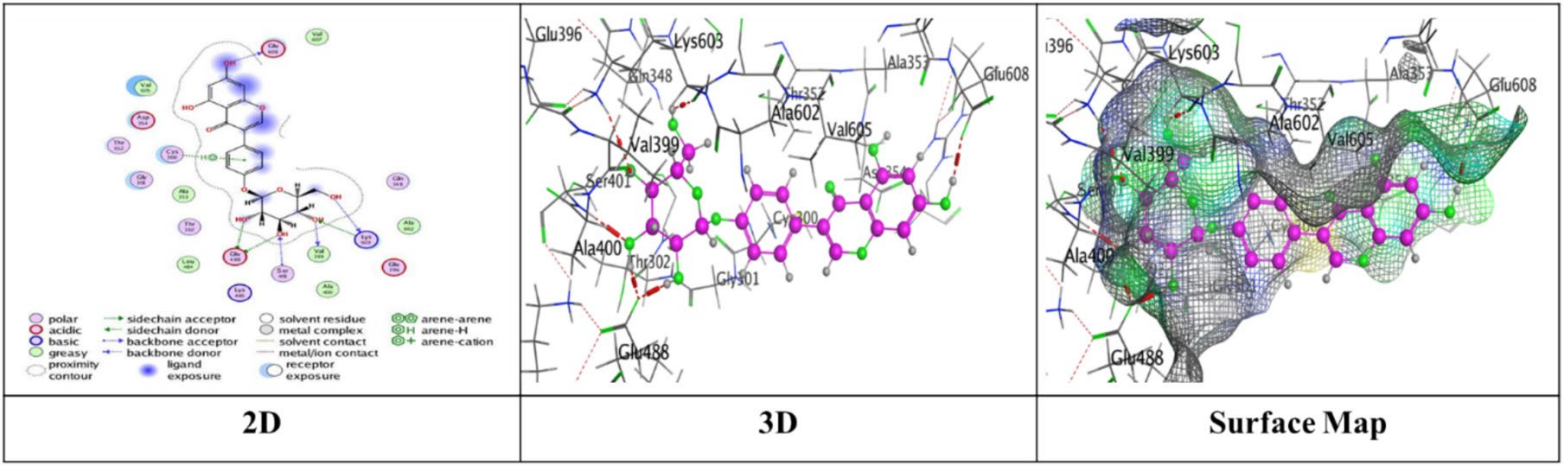
The interactions of compound **2** with active sites of (PDB ID: 1MOQ).

Meanwhile, compound **2** displayed five H-donor bonds, the first was raised between O18-atom of hydroxyl group with Glu 608 (2.91 Å), and two interactions from Glu 488 with O27-atom and O29-atom of hydroxyl group by an intermolecular distance (2.95 and 2.92Å), one O30-atom of hydroxyl group with Val 399, while the last attraction between O31-atom of hydroxyl group with Lys 603. Two H-acceptor bonds with Ser 401 and Lys 603. one π-H bond among Benzene ring with Cys 300 over binding scores, S = − 7.5991 with RMSD = 1.1785 (Fig. 7).

**Fig. 8:**
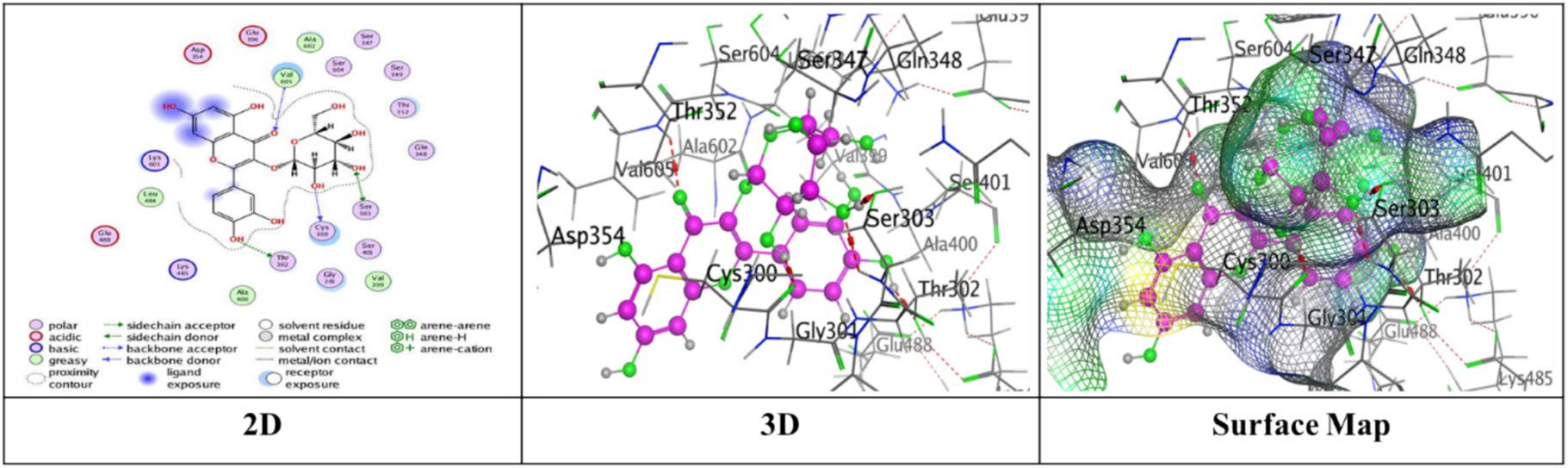
The interactions of compound **3** with active sites of (PDB ID: 1MOQ).

Furthermore, compound **3** exhibited a significant binding affinity (S = − 7.9167 kcal/mol) with an RMSD of 1.1720, resulting from three hydrogen bonds formed between the hydroxyl groups of O20, O29, and O31 atoms with Thr 302, Ser 303, and Cys 300, respectively. Two hydrogen-acceptor bonds: one between the O13-atom of the carbonyl group and Val 605 at an intermolecular distance of 3.25 Å, and the other between the O29-atom of the hydroxyl group and Ser 303 at an intermolecular distance of 3.11 Å (Fig. 8).

**Fig. 9:**
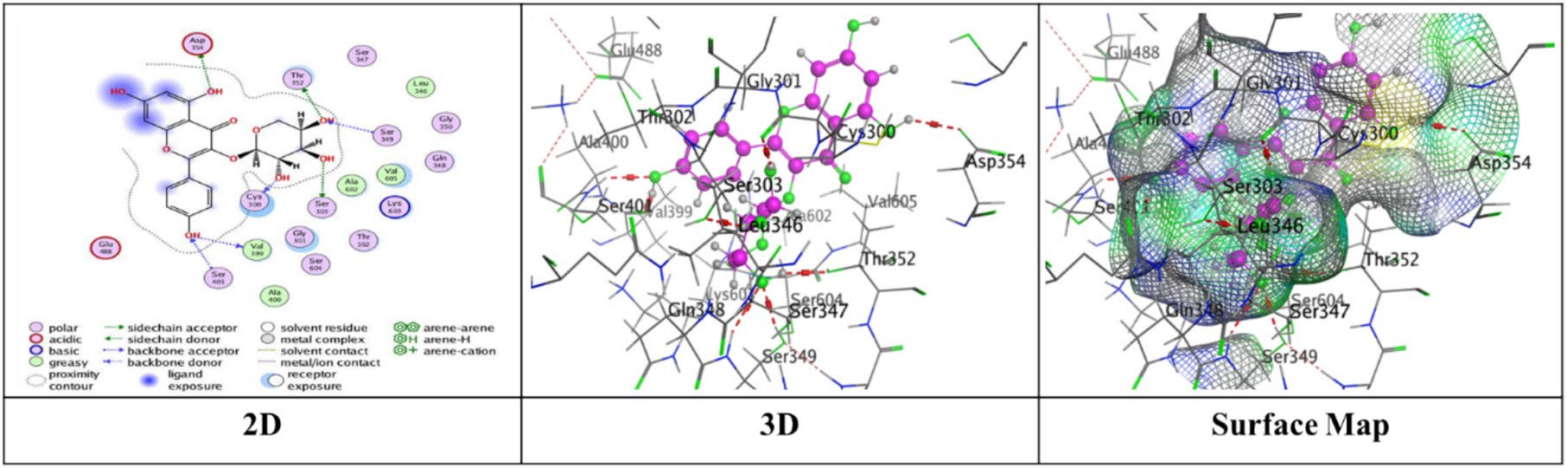
The interactions of compound **4** with active sites of (PDB ID: 1MOQ).

While compound **4** exhibited proper binding scores (S = − 7.2895 kcal/mol) through substantial interactions, including five hydrogen donor bonds with Val 399, Asp 354, Cys 300, Ser 303, and Thr 352, as well as three hydrogen acceptor bonds two with Ser 349 and one with Ser 401 over intermolecular distances (3.32, 2.98, 3.05 Å), respectively resulting in a binding score of S = − 7.2895 kcal/mol and an RMSD of 1.8506 (Fig. 9).

**Fig. 10:**
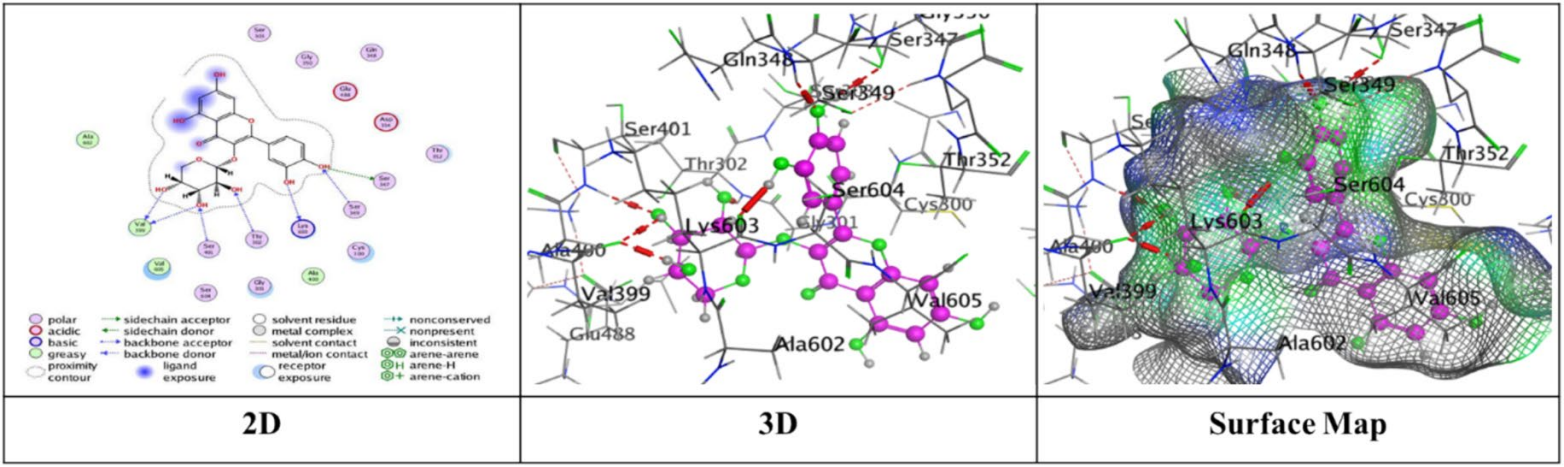
The interactions of compound **5** with active sites of (PDB ID: 1MOQ).

Further, compound **5** with energy score (S = − 7.0972 kcal/mol) formed four H-donor interactions, two amongst Val 399 with O29-atom and O30-atom of hydroxyl group, one between O18-atom with Ser 34, and O30-atom with Thr 352, Three H-acceptor bonds with Ser 349, Thr 302, and Ser 401 over intermolecular distances (2.84, 3.11, 2.88 Å), respectively over RMSD of 1.8358 (Fig. 10).

**Fig. 11:**
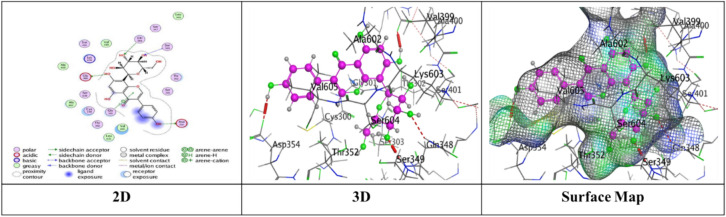
The interactions of compound **6** with active sites of (PDB ID: 1MOQ).

Compound **6** displayed three types of interaction, two H-donor bond, two H-acceptor bonds with oxygen atoms of hydroxyl groups with Glu 488 (2.84 Å), Asp 354 (2.95Å), Gln 348, (3.39 Å) and Ser 349 (3.21Å), respectively and other π–H interaction was presented through binding from Pyrone ring with Gly 301 (4.44 Å) through respectable docking score equal (S = − 7.7457 kcal/mol) (Fig. 11).

**Fig. 12:**
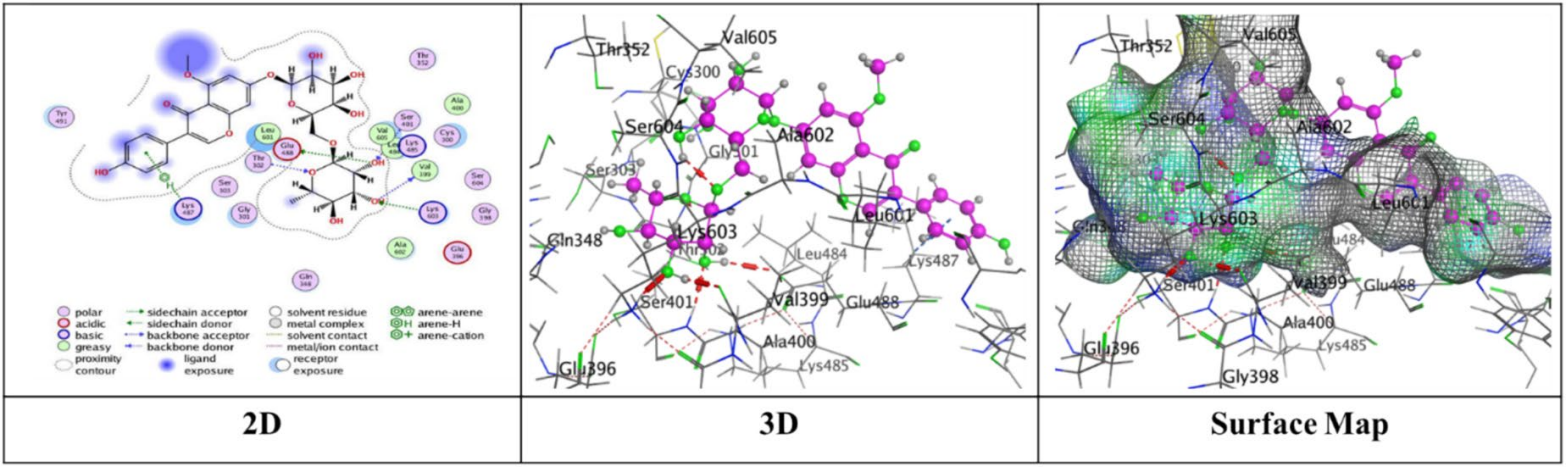
The interactions of compound **7** with active sites of (PDB ID: 1MOQ).

Finally, compound **7** demonstrated a superior binding affinity (S = − 8.6119 kcal/mol) with an RMSD of 1.7515. The optimal binding was ascribed to the elevated binding energy, alongside a commendable RMSD value, resulting from six interactions: two hydrogen donor bonds involving the O29-atom of the hydroxyl group with Glu 488 (3.25 Å) and Val 399 (2.72 Å), three hydrogen acceptor bonds with Thr 302 (3.29 Å), Ser 401 (3.06 Å), and Lys 603 (3.01 Å), as well as a π–H interaction from the benzene ring with Lys 487 (3.94 Å) (Fig. 12).

**Fig. 13:**
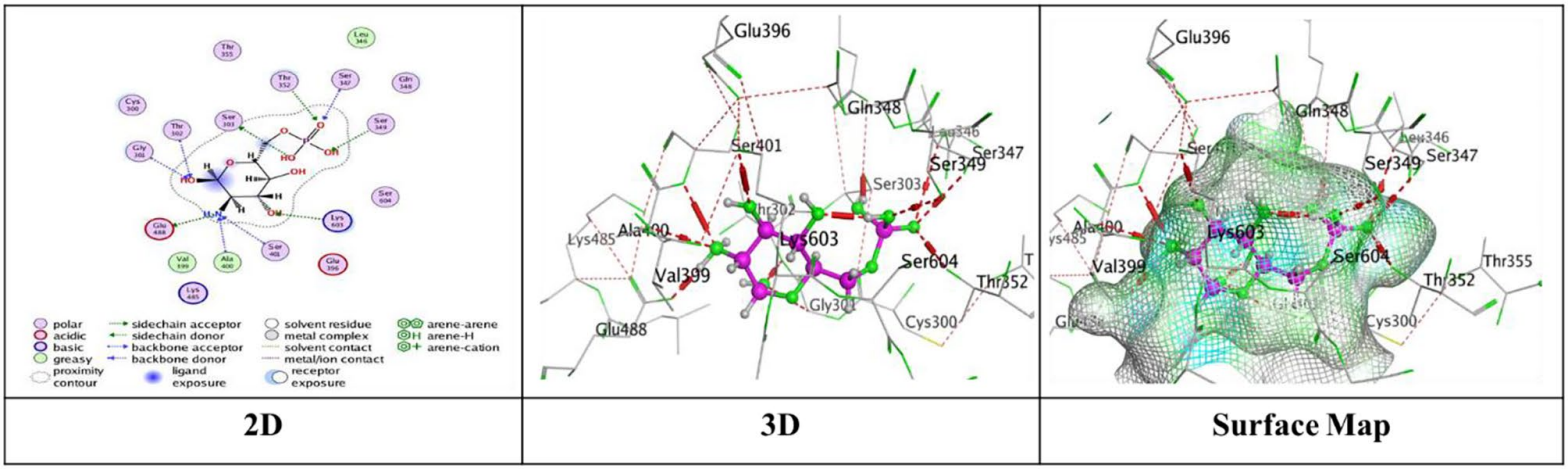
The interactions of Glucosamine-6-phosphate (GLP) with active sites of (PDB ID: 1MOQ).

Moreover, the standard compound used for docking was Glucosamine-6-phosphate (GLP), which was re-docked with the target protein (PDB ID: 1MOQ). The interaction analysis revealed that GLP formed multiple key interactions within the binding site, including two hydrogen bond donor interactions and nine hydrogen bond acceptor interactions, resulting in an RMSD of 1.3872 Å, which indicates a highly reliable and accurate docking protocol as reported in Table 3 (Fig. 13). The integration of (DFT) calculations alongside molecular docking provides a more comprehensive understanding of the bioactive potential of the synthesized compounds. While molecular docking predicts the binding affinities and interaction profiles of the ligands with target proteins, DFT offers valuable insights into the electronic structure, stability, and reactivity of the molecules. Key descriptors such as HOMO–LUMO energy gap, dipole moment, and (MEP) maps help identify electrophilic and nucleophilic regions, which may correspond to interaction sites observed in docking.

### Structure–activity relationship (SAR)

The antimicrobial potential of compounds **1–7** was evaluated through a combination of DFT calculations and molecular docking to explore how structural and electronic variations influence biological activity. Among these, compound **7** emerged as the most promising, displaying the lowest HOMO–LUMO energy gap (ΔE = 1.643 eV), highest dipole moment (7.562 D), and lowest global hardness (η = 0.821 eV), all indicative of high chemical reactivity, strong polarity, and enhanced interaction potential with biological targets. Its (MEP) map revealed favorable charge distribution for hydrogen bonding and electrostatic interactions. In contrast, compound **6** showed the least desirable electronic features, including the highest ΔE (2.270 eV) and lowest dipole moment (6.907 D), correlating with its weaker biological activity. Compounds **2–5** exhibited intermediate behavior, with moderate reactivity and binding scores. Notably, docking results confirmed the superior binding of compound **7** to GlcN-6-P synthase (PDB ID: 1MOQ), forming stable hydrogen bonds and π-interactions with residues such as Ser303 and Gln348, supported by a strong binding score (S = − 8.6119 kcal/mol). Overall, the SAR analysis highlights that enhanced antimicrobial activity is associated with low energy gaps, high electrophilicity and polarity, and the presence of functional groups enabling hydrogen bonding characteristics most evident in compound **7**, positioning it as a promising lead for further development.

### Antimicrobial activity assessment

**Fig. 14:**
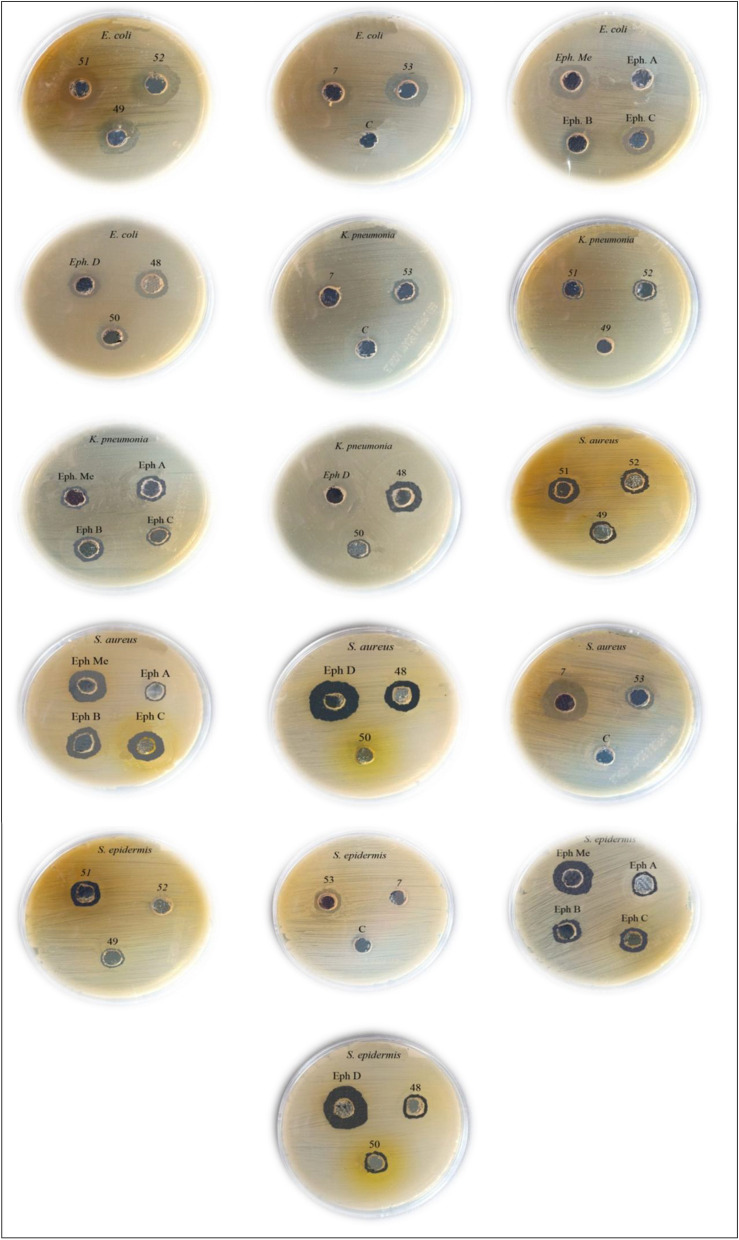
Antibacterial activities of *E. paralias* fractions and isolated compounds against different microbial strains.

The results presented in Table 4 demonstrate that compounds **1**, **4**, and **7**, along with the crude methanol extract and fractions Eph B and Eph C, exhibited broad-spectrum antimicrobial activity against all tested microbial strains (Fig. 14). Additionally, compounds **2**, **3**, and **5**, as well as fractions Eph A and Eph D, displayed antimicrobial effects against approximately 80% of the examined strains. Compared to the standard antibiotic gentamicin, the tested fractions and isolated compounds exhibited varying degrees of antimicrobial potency, suggesting their potential as promising candidates for the development of novel antimicrobial agents.

**Table 4:** The inhibition zone in mm of the extracts of *Ephorbia paralias* and seven isolated compounds compared to standard antibiotic.

Compounds/fractions	*Escherichia coli*		*Klebsiella pneumonia*		*Staphylococcus aureus*		*Staphylococcus epidermis*		*Bacillus subtilis*	
	Diameter of inhibition zone (in mm)	% Activity index	Diameter of inhibition zone (in mm)	% Activity index	Diameter of inhibition zone (in mm)	% Activity index	Diameter of inhibition zone (in mm)	% Activity index	Diameter of inhibition zone (in mm)	% Activity index
**1**	14.0	56	17.2	86	14.0	56	11.5	43	14.5	47
**2**	16	64	NA	–	13.0	52	10.0	37	16.0	52
**3**	12.7	51	10.0	50	NA	–	11.0	41	16.0	52
**4**	14.0	56	10.0	50	16.0	64	13.0	48	17.0	55
**5**	18.0	72	11.0	55	14.0	56	NA	–	14.0	45
**6**	12.5	50	NA	–	16.6	66	NA	–	NA	–
**7**	19.0	76	13.0	65	12.0	48	12.3	45	13.0	42
Eph Me_**crd**_	16.0	64	13.0	65	17.1	68	16.0	59	12.0	39
Eph A	NA	–	15.0	75	10.0	40	10.0	37	10.0	32
Eph B	14.0	56	16.0	80	14.0	56	13.0	48	13.0	42
Eph C	15.0	60	12.0	60	16.0	64	14.0	52	13.0	42
Eph D	12.0	48	NA	–	18.0	72	17.0	63	13.0	42
Gentamicin	25	100	20	100	25	100	27	100	31.0	100

Regarding *E. coli*, compounds **7**, and **5** showed the strongest activities with inhibition zones of 19.0, and 18.0 mm and activity indexes of 76, and 72% respectively, followed by compound **2**, Eph Me_crd_, and Eph C fraction with inhibition zones of 16.0, 16.0, and 15.0 mm and activity indexes 64, 64, and 60% respectively. Compounds **1**, **4**, and Eph B showed similar activities with an inhibition zone of 14.0 mm and an activity index of 56%. Compounds **3**, **6**, and Eph D showed the least activity than other compounds with inhibition zones of 12.7, 12.5, and 12.0 mm and activity indexes of 51, 50, and 48% respectively. Eph A fraction was resisted by *E. coli* microorganisms.

Regarding* K. pneumonia*, compound **1**, and Eph B fraction exhibited the highest antibacterial activity with inhibition zones of 17.2, and 16.0 mm, and activity indexes of 86, and 80% respectively, followed by Eph A fraction with an inhibition zone of 15.0 mm and activity index of 75%. Both compound **7** and the crude methanolic extract (Eph Mecrd) demonstrated comparable effects, showing inhibition zones of 13.0 mm and activity indices of 65%. The activities of compounds **3** and **4** were the least and similar with an inhibition zone of 10.0 mm and activity index of 50%. In contrast, compound **6** and the Eph D fraction showed no activity, indicating resistance of *K. pneumoniae* to these treatments. .

Regarding* Staphylococcus aureus*, Eph D fraction, Eph Me_crd_ extract and compound **6** exhibited the strongest activities with inhibition zones of 18.0, 17.1, 16.6 mm and activity indexes of 72, 68, 66% respectively. The Eph C fraction and compound **4** demonstrated similar levels of activity, with inhibition zones of 16.0 mm and an activity index of 64%. Compounds **1**, **5**, and Eph B showed identical activities with an inhibition zone of 14.0 mm and an activity index of 56%. The activities of compounds **2**, **7**, and Eph A fraction were the least with inhibition zones of 13.0, 12.0, and 10.0 mm and activity indexes of 52, 48, and 40% respectively. *Staphylococcus aureus* showed no resistance to the Euphorbia fractions and their isolated compounds.

Regarding* S. epidermis,* Eph D fraction exhibited the strongest activity with an inhibition zone of 17.0 mm and an activity index of 63%, followed by Eph C fraction with an inhibition zone of 14.0 mm and an activity index of 52%. The activities of compound **4**, and the Eph B fraction were similar with an inhibition zone of 13.0 mm and an activity index of 48%. Compounds **1** and **3** showed activities with inhibition zones of 11.5 and 11.0 mm and activity indexes of 43 and 41% respectively. The activities of compound **2**, and Eph A fraction were the least with an inhibition zone of 10.0 mm and an activity index of 37%. Compounds **5** and **6** were not effective against *S. epidermidis*.

Regarding *B. subtilis,* compound **4** showed the strongest activity with an inhibition zone of 17.0 mm and an activity index of 55%. The activities of compound **2**, and **3** were similar, with an inhibition zone of 16.0 mm and an activity index 52%, followed by compounds **1** and **5** which exhibited inhibition zones of 14.5 and 14.0 mm and activity indexes of 47 and 45% respectively. Fractions Eph B, Eph C, Eph D, and compound **7** were similar with an inhibition zone of 13.0 mm and an activity index of 42%. Eph Me_crd_ extract, and Eph A fraction exhibited the least activity with inhibition zones of 12.0, and 10.0 mm, and activity indexes of 39 and 32% respectively. Compounds **6** was resisted by *Bacillus subtilis*.

Our investigations of *Euphorbia paralias* revealed the presence of diverse flavonoids, terpenoids, and sterols, which may contribute to its broad-spectrum antimicrobial activity against both Gram-positive and Gram-negative pathogenic strains. Our anti-microbial results obtained from the *E. paralias* different fractions and purely separated compounds with a concentration of 2.5 mg/ml were listed in Table 4.

The broad antimicrobial spectrum exhibited by Eph Me_crd_, as well as fractions Eph B, and Eph C, suggests that these samples are rich in highly active and valuable compounds effective against both Gram-positive and Gram-negative bacteria. Fractions Eph B, and Eph C were the most active against *E. coli, K. pneumonia, S. aureus, S. epidermis* and *B. subtilis* with inhibition zones presented in Table 4, as a result of the presence of sterol **1** and flavonoid glycoside **4** which illustrate the broad spectrum activities of them.

Compound **1** exhibited broad-spectrum activity on both Gram-positive and Gram-negative bacteria, likely due to its ability to inhibit bacterial adhesion to fibronectin. This supports previous findings suggesting that structural modification of *β*-sitosterol may enhance its antibacterial efficacy, in accordance with the literature^31,68^.

Compound **4**, a kaempferol derivative bearing an arabinopyranoside moiety, exhibited antibacterial activity against *E. coli* with an inhibition zone of 14.0 mm by disruption of the bacterial cell membrane, leading to protein leakage into the extracellular environment. Inhibition of DNA gyrase of *E. coli* by kaempferol was another mechanism for its action^69^. It has the potential to reverse the resistance via inhibition of peptidoglycan and ribosome synthesis^70^. The inhibition zone of compound **4** against *S. aureus*, was 16.0 mm by inhibiting its DNA helicases as shown by Huang et al.^71^ Its activity against *B. subtilis* (inhibition zone of 17.0 mm) aligns with previous findings reported by Ghaffari et al.^72^, confirming its broad- spectrum of antibacterial activity against both Gram-positive and Gram-negative bacteria^73^.

Compounds **6, 2**, and **7** identified as isoflavone glycosides, exhibited antibacterial activity against *E. coli* with inhibition zones of 12.5, 16.0, 19.0 mm, and against *S. aureus* with inhibition zones of 16.6, 13.0, 16.0 mm, respectively. Their antibacterial mechanisms are likely related to concentration-dependent induction of cell death and membrane lysis, as supported by previous findings^74^.

Compound **7** contains a methoxyl group on ring A, which enhance its interactions that occur with the lipid bilayers of pathogenic microorganisms, thereby contributing to its antimicrobial activity^75^.

Flavonoid glycosides **3**, and **5**, which contain the quercetin aglycone, exert their antimicrobial effects by reducing bilayer thickness, damaging the cytoplasmic membrane, and causing the loss of intracellular components, leading to metabolic dysfunction and finally bacterial death^76–78^. Compounds **3** and **5** showed activities against *E. coli* with inhibition zones of 12.7, and 18.0 mm respectively, by inhibiting the biofilm formation of enteroaggregative *EAEC 042* strain of *E. coli*^79^.

### Insecticidal activity assessment

The toxicities of *E. paralias* Eph Me_crd_ extract, Eph A, Eph B, Eph C, Eph D fractions and isolated compounds **(1–7)** were examined and compared with the positive control azadirachtin (Okios 3.2% EC) against *A. gosspyii* and* A. biguttula*. Generally, The LC₅₀ values obtained for *E. paralias* fractions and isolated compounds against *Aphis gossypii* and *Amrasca biguttula* suggest moderate insecticidal activity^80^, demonstrating their potential as eco-friendly pest control agents compared with the control, as shown in Tables 5 and 6. For instance, the Eph D fraction exhibited the highest insecticidal efficacy than other fractions, with LC_50_ values of 391.04 and 341.25 ppm against *A. gosspyii* and *A. biguttula*, respectively. Also, the results demonstrated that the combination of compounds **2** and** 3** was the most efficacious against both* A. gosspyii* and* A. biguttula* with LC_50_ values of 397.39 and 332.93 ppm compared with Azadirachtin, respectively with LC₅₀ values below 100 ppm against various insect pests, indicating higher potency. Flavonoids such as genistein, quercetin and kaempferol have demonstrated significant potential in protecting crops from various insect pests, including *Spodoptera frugiperda, Helicoverpa zea, Bemisia tabaci* and* Aphis gossypii.* Flavonoids not only affect essential enzymes like acetylcholinesterase (AChE), but they also may affect neural channels in the nervous system of insects^81^. Maazoun et al. explained that flavonoid-rich extract inhibited several enzymes such as lipase, protease and *α*-amylase according to the concentration of the flavonoids^82^.

**Table 5:** Toxicity of *E. paralias* fractions and isolated compounds on cotton aphids (*A. gosspyii*) after 24 h under laboratory conditions.

Treatment	LC_50_ (ppm)	Confidence limit 95% (ppm)		LC_90_ (ppm)	Confidence limit 95% (ppm)		Slope ± S.E	Toxicity index (%) at LC_50_ value
		Lower	Upper		Lower	Upper		
Eph Me_crd_	676.65	560.63	810.74	1865.80	1391.57	3245.75	2.91 ± 0.51	57.79
Eph A	651.33	490.12	874.32	3145.90	2001.50	6923.12	1.87 ± 0.29	60.04
Eph B	408.94	306.58	546.98	1983.37	1269.11	4314.04	1.87 ± 0.29	95.62
Eph C	411.23	326.32	519.15	1486.18	984.68	3682.94	2.30 ± 0.47	95.09
Eph D	391.04	318.34	473.11	1159.44	845.86	2140.56	2.72 ± 0.49	100
1	747.83	625.99	903.22	2034.06	1496.05	3683.13	2.95 ± 0.52	3.66
2 and 3	397.39	320.66	487.76	1268.54	894.69	2581.39	2.54 ± 0.48	6.89
4	467.34	376.37	562.02	1383.15	1013.72	2596.72	2.72 ± 0.52	5.86
5	510.74	415.88	616.28	1502.59	1103.84	2721.65	2.73 ± 0.49	5.36
6	462.33	371.37	598.78	1673.10	1076.00	4551.75	2.29 ± 0.48	5.93
7	524.81	436.42	622.96	1385.05	1056.24	2273.97	3.04 ± 0.51	5.22
Azadirachtin (Okios 3.2% EC)	27.40	17.56	45.64	178.28	83.30	2003.89	1.576 ± 0.44	100

**Table 6:** Toxicity of *E. paralias* fractions and isolated compounds on cotton jassid (*A. biguttula*) after 24 h under laboratory conditions.

Treatment	LC_50_ (ppm)	Confidence limit 95% (ppm)		LC_90_ (ppm)	Confidence limit 95% (ppm)		Slope ± *S.E*	Toxicity index (%) at LC_50_ value
		Lower	Upper		Lower	Upper		
Eph Me_crd_	548.27	461.28	648.37	1529.05	1179.37	2331.42	2.88 ± 0.40	62.24
Eph A	548.14	405.71	731.91	2739.75	1757.35	2077.48	1.83 ± 0.29	62.26
Eph B	368.49	274.50	489.69	1779.81	1153.88	3767.03	1.87 ± 0.29	92.61
Eph C	369.28	294.98	450.29	1172.57	840.88	2277.04	2.55 ± 048	92.41
Eph D	341.25	273.99	409.14	984.63	742.74	1661.48	2.78 ± 0.48	100
1	606.50	489.35	731.23	1801.78	1330.45	3219.69	2.71 ± 0.48	3.39
2 and 3	332.93	264.48	400.94	990.33	741.45	1706.49	2.71 ± 0.48	6.17
4	493.52	397.85	597.22	1494.11	1091.83	2747.95	2.66 ± 048	4.16
5	521.45	426.56	628.82	1516.38	1114.91	2739.95	2.76 ± 0.49	3.94
6	477.51	384.93	574.65	1406.44	1044.80	2472.48	2.73 ± 0.48	4.30
7	513.09	431.73	599.79	1235.68	977.33	1856.24	3.36 ± 0.53	4.00
Azadirachtin (Okios 3.2% EC)	20.54	15.10	28.03	112.64	68.90	273.86	1.73 ± 0.28	100

## Conclusions

This study successfully integrates phytochemical profiling, quantum chemical calculations, and molecular docking analyses to explore the bioactive constituents of *Euphorbia paralias*. A total of seven flavonoids were isolated and structurally elucidated, while GC/MS analysis revealed 34 and 13 additional compounds from hexane and chloroform fractions, respectively. The methanol extract, various fractions, and isolated compounds demonstrated notable antimicrobial and insecticidal activities. Specifically, compounds **1**, **4**, and **6**, along with the chloroform and ethyl acetate fractions, exhibited broad-spectrum antibacterial effects, while the butanol fraction and a combination of compounds **2** and **3** showed promising insecticidal effects against common agricultural pests. Quantum chemical (DFT) calculations provided insights into the electronic characteristics linked to biological activity. Notably, compound 7 exhibited the lowest HOMO–LUMO gap (1.643 eV), highest dipole moment (7.562 Debye), and lowest chemical hardness (0.821 eV), suggesting its high reactivity and potential bioactivity. Molecular docking simulations further confirm strong binding affinities of the active compounds with microbial and insecticidal target proteins, reinforcing their pharmacological and pesticidal potential. The alignment between theoretical and experimental data reinforces the use of DFT and molecular docking as predictive tools in natural product research offering valuable insights for natural product-based drug discovery and eco-friendly pest managements. This study emphasize the potential of *Euphorbia paralias* as a promising source of novel antimicrobial agents and environmentally friendly insecticides. Further research on in vivo efficacy, toxicity assessments, and formulation development is recommended to translate these findings into practical pharmacological and agricultural applications.

## Supplementary Information

Supplementary Information.

## Data Availability

All data including experimental, spectroscopic, and computational data generated or analyzed during this study are included in this article and its Supplementary Information file. If any raw data files are required in another format, they can be obtained from the corresponding author upon reasonable request" in the manuscript.

## References

[1] James, T. A. & Harden, G. J. *Euphorbia paralias. Plant NET-New South Wales Flora Online* (Royal Botanic Gardens & Domain Trust, Sydney Australia, 2008).

[2] Boyce, L. & Buckeridge, J. *The Terrestrial Plants of the Rickett’s Point Urban Sanctuary: Beaumaris Vic 3193* 73 (Greypath Productions, Beaumaris, 2018).

[3] Sayed, M. D. Contribution to the constituents of Egyptian *Euphorbia geniculata* Jacq. and *Euphorbia prostrate* Ait. I. Triterpenoids and related substances. II. Flavonoids. In *American Society of Pharmacognosy Meeting* (1976).

[4] Lewis, W. *An Experimental History Of Materia Medica* 4th edn, 504 (Johnson, J, London, 1971).

[5] Pliny the Elder. Naturalis historia 26:45. London. Cambridge. William Heinemann, M. A. Harvard University Press. 1949–1954.

[6] Liu, J., Wang, X., Yong, H., Kan, J. & Jin, C. Recent advances in flavonoid-grafted polysaccharides: Synthesis, structural characterization, bioactivities and potential applications. *Int. J. Biol. Macromol.* **116**, 1011–1025. 10.1016/j.ijbiomac.2018.05.149 (2018). 29800657 10.1016/j.ijbiomac.2018.05.149

[7] Camero, C. M. et al. Anti-angiogenic activity of iridoids from *Galium tunetanum*. *Braz. J. Pharm. Sci.* **28**, 374–377. 10.1016/j.bjp.2018.03.010 (2018).

[8] Patel, K., Kumar, V., Rahman, M., Verma, A. & Patel, D. K. New insights into the medicinal importance, physiological functions and bioanalytical aspects of an important bioactive compound of foods ‘Hyperin’: Health benefits of the past, the present, the future. *Beni-Suef Univ. J. Basic appl. Sci.* **7**(1), 31–42. 10.1016/j.bjbas.2017.05.009 (2018).

[9] Zamora-Ros, R. et al. Dietary flavonoids, lignans and colorectal cancer prognosis. *Sci. Rep.* **5**, 14148. 10.1038/srep14148 (2015). 26369380 10.1038/srep14148 PMC4572925

[10] Avtar, C. & Bhawna, G. Chemistry and Pharmacology of flavonoids—a review. *Indian J. Pharm. Educ. Res.* **53**, 8–20. 10.5530/ijper.53.1.3 (2019).

[11] Reale, G. et al. Association between dietary flavonoids intake and prostate cancer risk: A case-control study in Sicily. *Complement. Ther. Med.* **39**, 14–18. 10.1016/j.ctim.2018.05.002 (2018). 30012385 10.1016/j.ctim.2018.05.002

[12] Rienks, J., Barbaresko, J. & Nothlings, U. Association of isoflavone biomarkers with risk of chronic disease and mortality: A systematic review and meta-analysis of observational studies. *Nutr. Res. Rev.* **75**(8), 616–641. 10.1093/nutrit/nux021 (2017). 10.1093/nutrit/nux021 28969363

[13] Ghani, A. S. A., El-Toumy, S. A., El-Dougdoug, W. I. A., Badr, W. H. & Hassan, H. M. New acetyl triterpenoidal and biological activities of *Euphorbia Paralias* and *Euophorbia Geniculata* (Euphorbiaceae) from Egypt. *Egypt. J. Chem.* **63**(10), 3583–3595. 10.21608/ejchem.2020.20211.2215 (2020).

[14] Jakupovic, J., Morgenstern, T., Marco, J. A. & Berendsohn, W. Diterpenes from *Euphorbia paralias*. *Phytochem.* **47**(8), 1611–1619. 10.1016/S0031-9422(97)00832-7 (1998).

[15] Marco, J. A., Sanz-Cervera, J. F., Yuste, A., Jakupovic, J. & Lex, J. Terracinolides A and B, two bishomoditerpene lactones with a novel carbon framework from *Euphorbia terracina*. *J. Org. Chem.* **61**(5), 1707–1709. 10.1021/jo951910f (1996). 11667040 10.1021/jo951910f

[16] Öksüz, S. et al. Paralinones A and B, novel diterpene esters from *Euphorbia paralias*. *Tetrahedron* **53**(9), 3215–3222. 10.1016/S0040-4020(97)00032-X (1997).

[17] Abdelgaleil, S. A. M., Kassem, M. I., Doe, M., Baba, M. & Nakatani, M. Diterpenoids from *Euphorbia paralias*. *Phytochem.* **58**, 1135–1139. 10.1016/s0031-9422(01)00393-4 (2001). 10.1016/s0031-9422(01)00393-4 11730879

[18] Schirmer, S., Sengonca, C. & Blaeser, P. Inflence of abiotic factors on some biological and ecological characteristics of the aphid parasitoid *Aphelinus asychis* (Hymenoptera: Aphelinidae) parasitizing *Aphis gossypii* (Sternorrhyncha: Aphididae). *Eur. J. Entomol.* **105**, 121–129. 10.14411/eje.2008.017 (2008).

[19] Singh, A., Singh, J. J., Singh, K. & Rani, P. Host range and biology of *Amrasca biguttula* (Hemitpera: Cicadellidae). *Int. J. Environ. Ecol. Fam. Urban Stud.* **8**, 19–24. 10.24247/ijeefusapr20183 (2018).

[20] Boulos, L. *Flora of Egypt* Vol. 2, 288 (Al Hadara Publishing, Cairo, 2000).

[21] Balouiri, M., Sadiki, M. & Ibnsouda, S. K. Methods for in vitro evaluating antimicrobial activity: A review. *J. Pharm. Anal.* **6**(2), 71–79. 10.1016/j.jpha.2015.11.005 (2016). 29403965 10.1016/j.jpha.2015.11.005 PMC5762448

[22] Elhefni, M. A., El-Rokh, A. R., El-Rafey, H. H., Tadros, L. K. & Taher, M. A. Phytochemical profiling and isolation of bioactive polyphenols from *Ipomoea carnea*. *Egypt. J. Chem.* **66**(12), 529–543. 10.21608/ejchem.2023.189242.7495 (2023).

[23] Abbott, W. S. A method of computing the effectiveness of an insecticide. *J. Econom. Entom.* **18**(2), 265–267. 10.1093/jee/18.2.265a (1925).

[24] Finney, D. J. *Probit Analysis, a Statistical Treatment of the Sigmoid Response Curve* 7th edn, 333 (Cambridge University Press, Cambridge, 1950).

[25] Sun, Y. P. Toxicity index, an improved method of comparing the relative toxicity of insecticides. *J. Econom. Entom.* **43**(1), 45–53. 10.1093/jee/43.1.45 (1950).

[26] Elmorsy, M. R., Abdel-Latif, E., Gaffer, H. E., Badawy, S. A. & Fadda, A. A. Theoretical studies, anticancer activity, and photovoltaic performance of newly synthesized carbazole—based dyes. *J. Mol. Struct.* **1255**(2), 132404. 10.1016/j.molstruc.2022.132404 (2022).

[27] Purawarga Matada, G. S. et al. Molecular docking and molecular dynamic studies: Screening of phytochemicals against EGFR, HER2, estrogen and NF-KB receptors for their potential use in breast cancer. *J. Biomol. Struct. Dyn.* **40**(13), 6183–6192. 10.1016/j.molstruc.2022.132404 (2022). 33525984 10.1080/07391102.2021.1877823

[28] Sajid, A. et al. In-vitro and molecular docking studies of plant secondary metabolites isolated from *Hypericum oblongifolium* as antibacterial agents and lipoxygenase (5-LOX) inhibitors. *J. Mol. Struct.* **1312**, 138549 (2024).

[29] Kasal, A., Budesinsky, M. & Griffiths, W. J. Spectroscopic methods of steroid analysis. In *Steroid Analysis* (eds Makin, H. L. J. & Gower, D. B.) 102 (Springer, New York, 2010).

[30] Li, L. Z., Wang, M. H., Sun, J. B. & Liang, J. Y. Flavonoids and other constituents from *Aletris spicata* and their chemotaxonomic significance. *Nat. Prod. Res.* **28**(15), 1214–1217. 10.1080/14786419.2014.921918 (2014). 24896299 10.1080/14786419.2014.921918

[31] Tagne, R. S. et al. Anticancer and antioxidant activities of methanol extracts and fractions of some Cameroonian medicinal plants. *Asian Pac. J. Trop. Med.* **7**, S442–S447. 10.1016/S1995-7645(14)60272-8 (2014). 10.1016/S1995-7645(14)60272-8 25312165

[32] Wang, Y. et al. Study of steroidal saponins in *Dioscorea zingiberensis* C.H. Wright. *J. Nat. Prod.* **2**, 123–132 (2009).

[33] Bilia, A. R., Mendez, J. & Morelli, I. Phytochemical investigations of *Licania*. genus Flavonoids and triterpenoids from Licania carii. *Pharm. Act. Helv.* **71**(3), 191–197. 10.1016/0031-6865(96)00010-6 (1996).

[34] Li, D. et al. Chemical constituents of the whole plants of *Houttuynia Cordata*. *Chem. Nat. Compd.* **53**(2), 365–367. 10.1007/s10600-017-1991-6 (2017).

[35] Lima, L. D. et al. Anti–Zika virus activity and isolation of flavonoids from ethanol extracts of *Curatella americana* L. Leaves. *Molecules* **28**(6), 2546. 10.3390/molecules28062546 (2023). 36985517 10.3390/molecules28062546 PMC10054362

[36] Chen, G. et al. Chemical constituents from Rhododendron spinuliferum. *Chem. Nat. Compd.* **45**(5), 725–727. 10.1007/s10600-009-9410-2 (2009).

[37] Lee, Y. E. et al. Flavonoids from *Woodfordia fruticosa* as potential SmltD inhibitors in the alternative biosynthetic pathway of peptidoglycan. *Bioorg. Med. Chem. Lett.* **36**(3), 127787. 10.1016/j.bmcl.2021.127787 (2021). 33460740 10.1016/j.bmcl.2021.127787

[38] Kamel, A. I., Elfedawy, M. G., ElShamy, M. M. & Abdel-Mogib, M. New physiologically active Kaempferol Glucoside from *Abutilon pannosum*. *J. Sci. Eng. App.* **6**(3), 78–87 (2017).

[39] Chen, G. et al. Chemical constituents from *Rhododendron spinuliferum*. *Chem. Nat. Compd.* **45**(5), 725–727. 10.1007/s10600-009-9410-2 (2009).

[40] Fraisse, D., Heitz, A., Carnat, A., Carnat, A. P. & Lamaison, J. L. Quercetin 3-arabinopyranoside, a major flavonoid compound from *Alchemilla xanthochlora*. *Fitoterapia* **71**(4), 463–464. 10.1016/S0367-326X(00)00145-3 (2000). 10925029 10.1016/s0367-326x(00)00145-3

[41] Rodrigues, C. F. et al. Evaluation of potential thrombin inhibitors from the white mangrove (*Laguncularia racemosa* (L.) C.F. Gaertn.). *Mar. Drugs* **13**(7), 4505–4519. 10.3390/md13074505 (2015). 26197325 10.3390/md13074505 PMC4515630

[42] Kinjo, J. et al. Studies on the constituents of *Pueraria lobate*. III: Isoflavonoids and related compounds in the roots and the voluble stems. *Chem. Pharm. Bull.* **35**, 4846–4850 (1987).

[43] Kamel, A. I., El-Rokh, A. R., Dawidar, A. M. & Abdel-Mogib, M. Bioactive compounds from *Retama raetam* (Forssk.) Webb & Berthel. and their insecticidal activity against cotton pests *Aphis gosspyii* and *Amrasca biguttula*. *Fitoterapia* **172**, 105749. 10.1016/j.fitote.2023.105749 (2024). 37972716 10.1016/j.fitote.2023.105749

[44] Cheng, J., Zhao, Y. Y., Wang, B., Qiao, L. & Liang, H. Flavonoids from *Millettia nitida* var. hirsutissima. *Chem. Pharm. Bull.* **53**(4), 419–421. 10.1248/cpb.53.419 (2005). 10.1248/cpb.53.419 15802842

[45] Gill, A. O. & Holley, R. A. Mechanisms of bactericidal action of cinnamaldehyde and eugenol against Listeria monocytogenes and *Lactobacillus sakei*. *Appl. Environ. Microbiol.* **70**(10), 5750–5755 (2004). 15466510 10.1128/AEM.70.10.5750-5755.2004 PMC522076

[46] Park, I. K., Shin, S. C., Kim, J. & Lee, S. G. Insecticidal activities of constituents identified in the essential oil from *Coriandrum sativum* seeds against *Callosobruchus chinensis* (L.) and *Sitophilus oryzae* (L.). *J. Stored Prod. Res.* **43**(3), 292–297 (2007).

[47] Ali, I. et al. Synthesis, biological evaluation and molecular docking studies of novel tetralone derivatives as anticancer agents. *Eur. J. Med. Chem.* **64**, 600–611 (2013).

[48] Marchese, A. et al. Antimicrobial and anti-inflammatory activities of eugenol and isoeugenol: A review. *Microb. Pathog.* **112**, 566–582 (2017).

[49] Syed, Z. & Leal, W. S. Acute olfactory response of Culex mosquitoes to a human- and bird-derived attractant. *Proc. Natl. Acad. Sci. U.S.A.* **105**(36), 13598–13603 (2008). 19858490 10.1073/pnas.0906932106 PMC2767364

[50] Dosoky, N. S. & Setzer, W. N. Chemical composition and biological activities of essential oils of Curcuma species. *Phytochem. Rev.* **17**(4), 745–772. 10.1007/s11101-018-9555-9 (2018). 10.3390/nu10091196 PMC6164907 30200410

[51] Dugasani, S. et al. Comparative antioxidant and anti-inflammatory effects of [6]-gingerol, [8]-gingerol, [10]-gingerol and [6]-shogaol. *J. Ethnopharmacol.* **127**(2), 515–520 (2010). 19833188 10.1016/j.jep.2009.10.004

[52] Jayawardena, T. U. et al. Loliolide, isolated from *Sargassum horneri*; abate LPS-induced inflammation via TLR mediated NF-κB MAPK pathways in macrophages. *Algal Res.* **56**, 102297 (2021).

[53] Ghimire, B. K. et al. Biological activities and chemical profiling of fatty acid methyl esters from medicinal plants. *Sci. Rep.* **11**(1), 13234 (2021).34168195

[54] Demetzos, C. & Dimas, K. S. Labdane-type diterpenes: Chemistry and biological activity. *Stud. Nat. Prod. Chem.* **25**, 235–292 (2001).

[55] Kim, J. H. et al. Anti-inflammatory and antinociceptive activities of sinapyl alcohol isolated from *Brassica juncea*. *Arch. Pharm. Res.* **31**(5), 658–664. 10.1007/s12272-001-1226-2 (2008).

[56] Niu, L. et al. 1-Monopalmitin promotes lung cancer cells apoptosis through PI3K/A kt pathway in vitro. *Environ. Toxicol.* **38**(11), 2621–2631 (2023). 37466199 10.1002/tox.23897

[57] Menendez, J. A. & Lupu, R. Mediterranean dietary traditions for the molecular treatment of human cancer: Anti-oncogenic actions of the main olive oil’s monounsaturated fatty acid (oleic acid). *Curr. Pharm. Biotechnol.* **7**(6), 495–502. 10.2174/138920106779116856 (2006). 17168666 10.2174/138920106779116900

[58] Okasha, N. I. et al. Identification of potential antiviral compounds from Egyptian sea stars against MERS–CoV with the in vitro and in silico experiments. *Nat. Prod. Res.* **22**, 1–7 (2024). 10.1080/14786419.2024.2335361 38563220

[59] Kusumah, D. et al. Linoleic acid, α-linolenic acid, and monolinolenins as antibacterial substances in the heat-processed soybean fermented with *Rhizopus oligosporus*. *Biosci. Biotechnol. Biochem.* **84**(6), 1285–1290 (2020). 32089087 10.1080/09168451.2020.1731299

[60] Ghosh, T., Maity, T. K. & Singh, J. Evaluation of antioxidant and antimicrobial activity of γ-sitosterol isolated from methanol extract of root bark of *Aegle marmelos* Corr. *J. Chem. Pharm. Res* **5**(2), 233–237 (2013).

[61] Mishra, P. et al. Biological characterization of α-tocospiro A from *Acalypha indica* and its in vitro antioxidant and anti-inflammatory potential. *Appl. Biochem. Biotechnol.* **195**(3), 1834–1850. 10.1007/s12010-023-04363-7 (2023). 10.1007/s12010-023-04363-7 36701094

[62] Lindor, K. D. et al. Ursodeoxycholic acid for primary biliary cirrhosis. *Hepatology* **50**(1), 308–315 (2009).

[63] Porter, F. D. et al. Cholestane-3β,5α,6β-triol and 7-ketocholesterol as biomarkers for Niemann-Pick C1 disease. *J. Lipid Res.* **51**(5), 1224–1235 (2010).

[64] Akinmoladun, F. O. et al. Antibacterial and anti-inflammatory activity of triterpenoids from *Euphorbia hirta*. *J. Herb. Med.* **22**, 100350 (2020).

[65] Alharbi, A. et al. Molecular modeling and antimicrobial activity of newly synthesized benzothiazolo [3, 2–a] pyrimidine clubbed thiazole derivatives. *Heliyon* **10**(19), e38905. 10.1016/j.heliyon.2024.e38905 (2024). 10.1016/j.heliyon.2024.e38905 PMC11492252 39435077

[66] Krishnan, K. G. et al. Hydrazide-integrated carbazoles: Synthesis, computational, anticancer and molecular docking studies. *New J. Chem.* **43**(30), 12069–12077 (2019).

[67] Sefrji, F. O. et al. Synthetic approaches for novel fused pyrimidine derivatives: Design, structural characterization, antiviral, antitumor, and molecular docking evaluation. *Heliyon*, **10**(24), e40903. 10.1016/j.heliyon.2024.e40903 (2024). 10.1016/j.heliyon.2024.e40903 PMC11667641 39720060

[68] Njinga, N. S. et al. Evaluation of antimicrobial and antioxidant activity of β-sitosterol-3-*O*-glucoside isolated from *Lannea kerstingii* Engl. & *K. Krause* (Anacardiaceae). *Int. J. Glob. Sustain.* **2**, 9 (2016).

[69] Wu, T., Zang, X., He, M., Pan, S. & Xu, X. Structure–activity relationship of flavonoids on their anti *escherichia coli* activity and inhibition of DNA gyrase. *J. Agric. Food Chem.* **61**, 8185–8190. 10.1021/jf402222v (2013). 23926942 10.1021/jf402222v

[70] Eumkeb, G., Siriwong, S., Phitaktim, S., Rojtinnakorn, N. & Sakdarat, S. Synergistic activity and mode of action of flavonoids isolated from smaller galangal and amoxicillin combinations against amoxicillin-resistant *Escherichia coli*. *J. Appl. Microbiol.* **112**, 55–64. 10.1111/j.13652672.2011.05190.x (2012). 22111967 10.1111/j.1365-2672.2011.05190.x

[71] Huang, Y. H., Huang, C. C., Chen, C. C., Yang, K. J. & Huang, C. Y. Inhibition of *Staphylococcus aureus* Pria Helicase by Flavonol Kaempferol. *Prot. J.* **34**, 169–172. 10.1007/s10930-015-9609-y (2015). 10.1007/s10930-015-9609-y PMC7088215 25894858

[72] Ghaffari, M. A. et al. Biological and phytochemical investigations of crude extracts of *Astragalus creticus*. *Pak. J. Pharm. Sci.* **34**, 403–409 (2021). 34275786

[73] Karimi, E., Jaafar, H. Z. & Ahmad, S. Phytochemical analysis and antimicrobial activities of methanolic extracts of leaf, stem and root from different varieties of *Labisa pumila* Benth. *Molecules* **16**(6), 4438–4450. 10.3390/molecules16064438 (2011). 21623314 10.3390/molecules16064438 PMC6264691

[74] Sadowska, K. H. et al. Genistein and gut inflammation: Role of nitric oxide. *Proc. Soc. Exp. Biol. Med.* **217**(1), 351–357. 10.3390/molecules18010322 (1998). 9492347 10.3181/00379727-217-44244

[75] Oteiza, P. I., Erlejman, A. G., Verstraeten, S. V., Keen, C. L. & Fraga, C. G. Flavonoid membrane Interactions: A protective role of flavonoids at the membrane surface?. *Clin. Dev. Immunol.* **12**(1), 19–25. 10.1080/10446670410001722168 (2005). 15712595 10.1080/10446670410001722168 PMC2270717

[76] Tagousop, C. N., Tamokou, J. D., Ekom, S. E., Ngnokam, D. & Voutquenne Nazabadioko, L. Antimicrobial activities of flavonoid glycosides from *Graptophyllum grandulosum* and their mechanism of antibacterial action. *BMC Complement. Altern. Med.* **18**, 252. 10.1186/s12906-018-2321-7 (2018). 30219066 10.1186/s12906-018-2321-7 PMC6139119

[77] Hartmann, M. et al. Damage of the bacterial cell envelope by antimicrobial peptides gramicidin S and PGLa as revealed by transmission and scanning electron microscopy. *Antimicrob. Agents Chemother.* **54**, 3132–3142. 10.1128/AAC.00124-10 (2010). 20530225 10.1128/AAC.00124-10 PMC2916356

[78] Djouossi, M. G. et al. Antimicrobial and antioxidant flavonoids from the leaves of *Oncoba spinosa* Forssk (Salicaceae). *BMC Complement. Altern. Med.* **15**, 134. 10.1186/s12906-015-0660 (2015). 25928352 10.1186/s12906-015-0660-1 PMC4424558

[79] Barboza, T. J. S., Ferreira, A. F., Ignácio, A. C. P. R. & Albarello, N. Cytotoxic, antibacterial and antibiofilm activities of aqueous extracts of leaves and flavonoids occurring in *Kalanchoe pinnata* (Lam.) Pers. *J. Med. Plants Res.* **10**(41), 763–770. 10.5897/JMPR2016.6260 (2016).

[80] World Health Organization. The WHO recommended classification of pesticides by hazard and guidelines to classification 2019. In *The WHO Recommended Classification of Pesticides by Hazard and Guidelines to Classification 2019* (2020).

[81] Pereira, V., Figueira, O. & Castilho, P. C. Flavonoids as insecticides in crop protection—a review of current research and future prospects. *Plants* **13**(6), 776. 10.3390/plants13060776 (2024). 38592833 10.3390/plants13060776 PMC10975847

[82] Maazoun, A. M. et al. Phytochemical profile and insecticidal activity of *Agave americana* Leaf extract towards (L.) (Coleoptera: Curculionidae). *Environ. Sci. Pollut. Res. Int.* **26**, 19468–19480. 10.1007/s11356-019-05316-6 (2019). 31077051 10.1007/s11356-019-05316-6

