# Supplementary material for: Phytochemical, biological, DFT, and molecular docking evaluation of Euphorbia paralias
Source: Sci Rep. 2025 May 23;15:17961. doi: 10.1038/s41598-025-02420-1 (PMC12102368; doi:10.1038/s41598-025-02420-1)
Supplement: Supplementary file 1 — Supplementary Information. [file 41598_2025_2420_MOESM1_ESM.pdf]

Supplementary materials

Chemical Constituents and Biological Activities of *Euphorbia paralias*: Phytochemical, Quantum Chemical, and Molecular Docking Studies

Abdullah I. Kamel[1,]*, Safa A. Badawy[3], Mamdouh Abdel-Mogib[3] & Ahmed Ramadan El-Rokh[2]

[1]Chemistry Department, Faculty of Science, New Mansoura University, Mansoura, Egypt.
[2]Plant Protection Research Institute, Agricultural Research Center, Giza-12618, Egypt.
[3]Chemistry Department, Faculty of Science, Mansoura University, Mansoura-35516, Egypt.

Table of Contents

Figure S1: ^{1}H NMR (500 MHz) spectrum of *β*-sitosterol-3-*O*-*β*-D-glucoside (1) in C_5D_5N
Figure S1a: Expanded ^{1}H NMR (500 MHz) spectrum of *β*-sitosterol-3-*O*-*β*-D-glucoside (1) in C_5D_5N
Figure S1b: Expanded ^{1}H NMR (500 MHz) spectrum of *β*-sitosterol-3-*O*-*β*-D-glucoside (1) in C_5D_5N
Figure S2: ^{13}C NMR (125 MHz) spectrum of *β*-sitosterol-3-*O*-*β*-D-glucoside (1) in C_5D_5N
Figure S2a: Expanded ^{13}C NMR (125 MHz) spectrum of *β*-sitosterol-3-*O*-*β*-D-glucoside (1) in C_5D_5N
Figure S2b: Expanded ^{13}C NMR (125 MHz) spectrum of *β*-sitosterol-3-*O*-*β*-D-glucoside (1) in C_5D_5N

Figure S3: ^{1}H NMR (500 MHz) spectrum of genistein-4'-*β*-*O*-D-glucopyranoside (2) and quercetin-3-*β*-*O*-D-glucoside (3) in CD_3OD
Figure S3a: Expanded ^{1}H NMR (500 MHz) spectrum of genistein-4'-*β*-*O*-D-glucopyranoside (2) and quercetin-3-*β*-*O*-D-glucoside (3) in CD_3OD

Figure S4: ^{1}H NMR (500 MHz) spectrum of kaempferol-3-*O*-*α*-D-arabinopyranoside (4) in DMSO-*d6*
Figure S4a: Expanded ^{1}H NMR (500 MHz) spectrum of kaempferol-3-*O*-*α*-D-arabinopyranoside (4) in DMSO-*d6*

Figure S5: ^{1}H NMR (500 MHz) spectrum of quercetin-3-*O*-*α*-D-arabinopyranoside (5) in DMSO-*d6*
Figure S5a: Expanded ^{1}H NMR (500 MHz) spectrum of quercetin-3-*O*-*α*-D-arabinopyranoside (5) in DMSO-*d6*

Figure S6: ^{1}H NMR (400 MHz) spectrum of genistein-8*β*-*C*-glucoside (6) in DMSO-*d*6
Figure S6a: Expanded ^{1}H NMR (400 MHz) spectrum of genistein-8*β*-*C*-glucoside (6) in DMSO-*d*6
Figure S7: APT ^{13}C NMR (100 MHz) spectrum of genistein-8*β*-C-glucoside (6) in DMSO-*d6*
Figure S8: Edited HSQC spectrum of genistein-8*β*-C-glucoside (6) in DMSO-*d*6 in DMSO-*d6*

Figure S9: ^{1}H NMR (500 MHz) spectrum of hirsutissimiside B (7) in CD_3OD
Figure S9a: Expanded ^{1}H NMR (500 MHz) spectrum of hirsutissimiside B (7) in CD_3OD

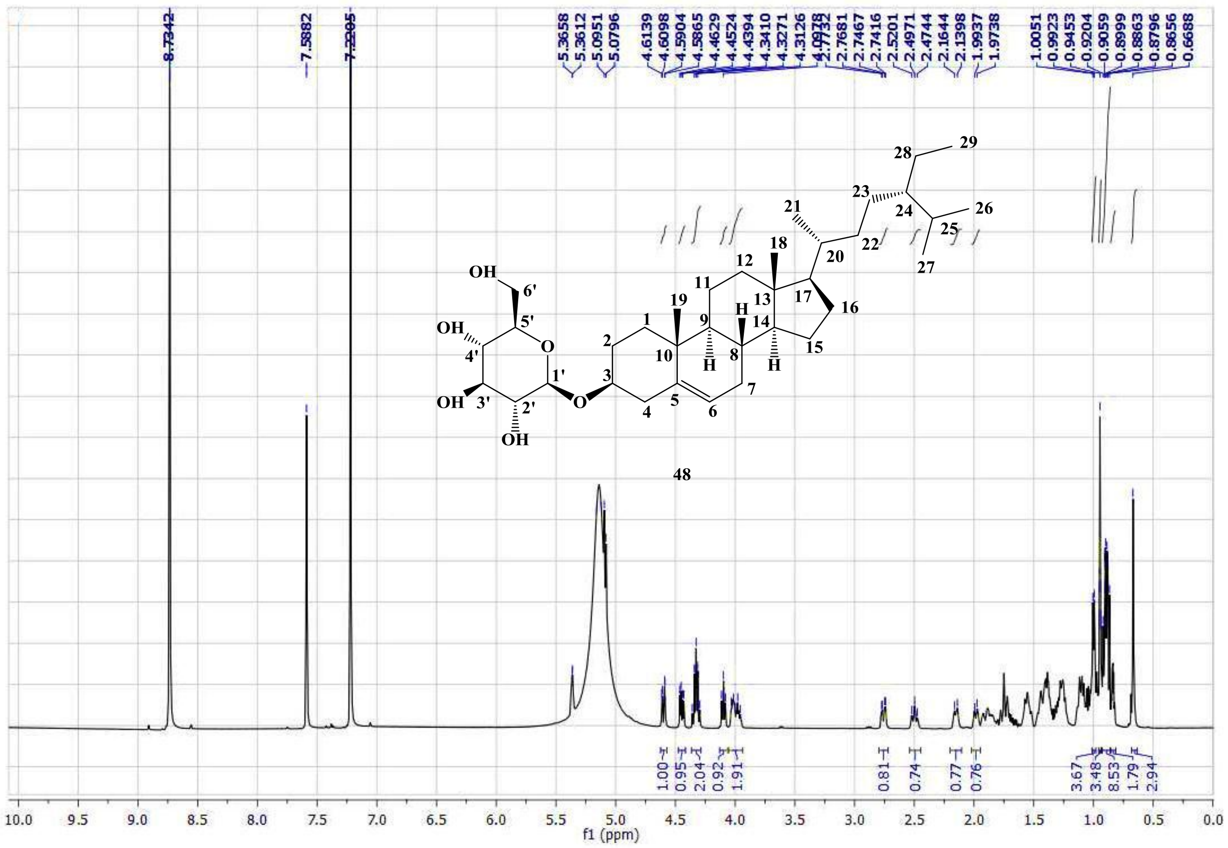

Figure S1: ^{1}H NMR (500 MHz) spectrum of *β*-sitosterol-3-*O*-*β*-D-glucoside (1) in C_5D_5N

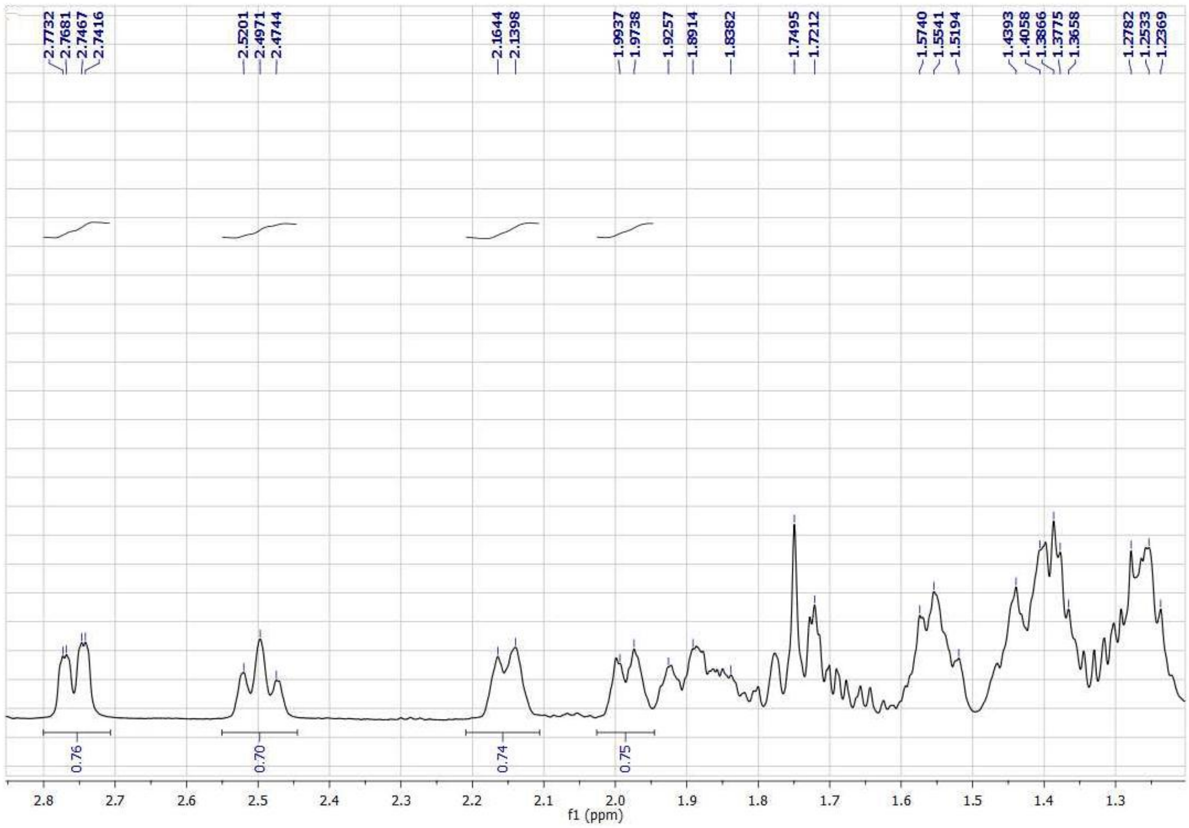

Figure S1a: Expanded ^{1}H NMR (500 MHz) spectrum of *β*-sitosterol-3-*O*-*β*-D-glucoside (1) in C_5D_5N

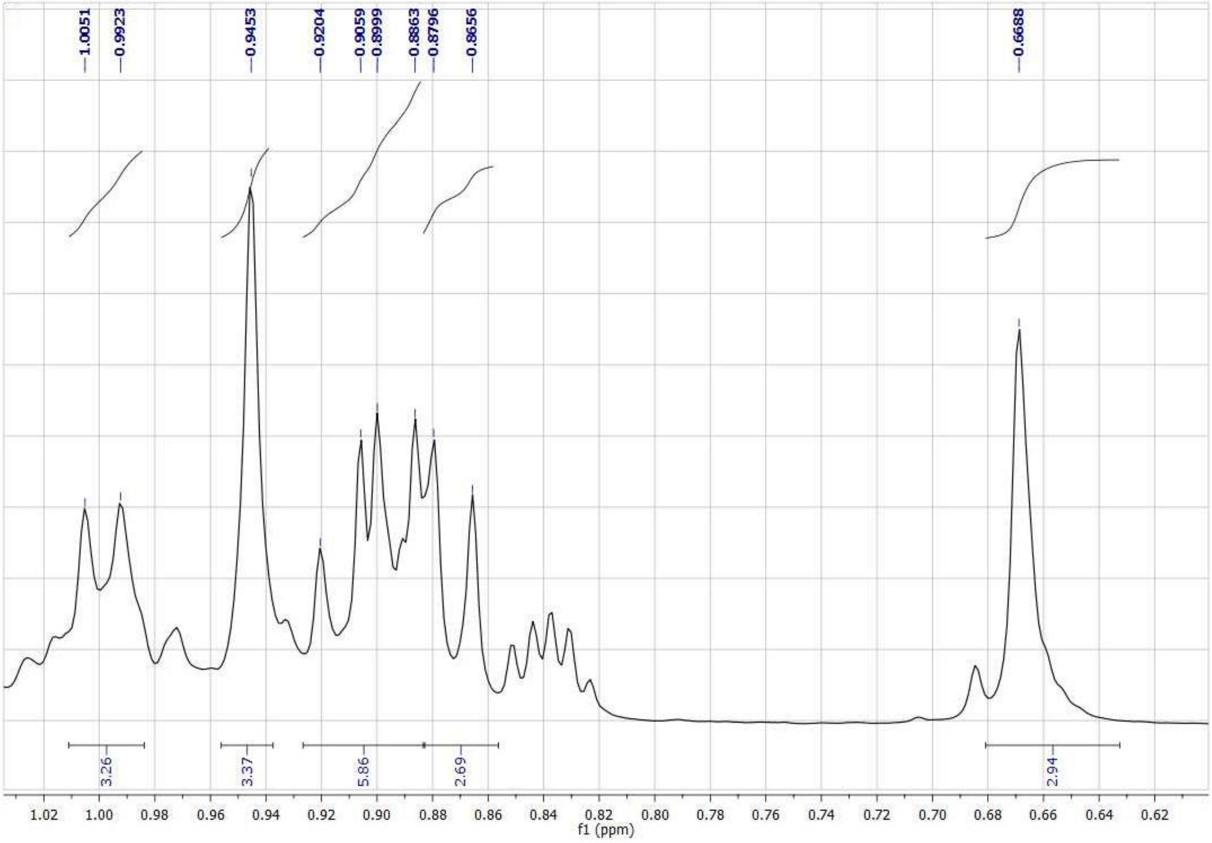

Figure S1b: Expanded ¹H NMR (500 MHz) spectrum of *β*-sitosterol-3-*O*-*β*-D-glucoside (1) in C_5D_5N

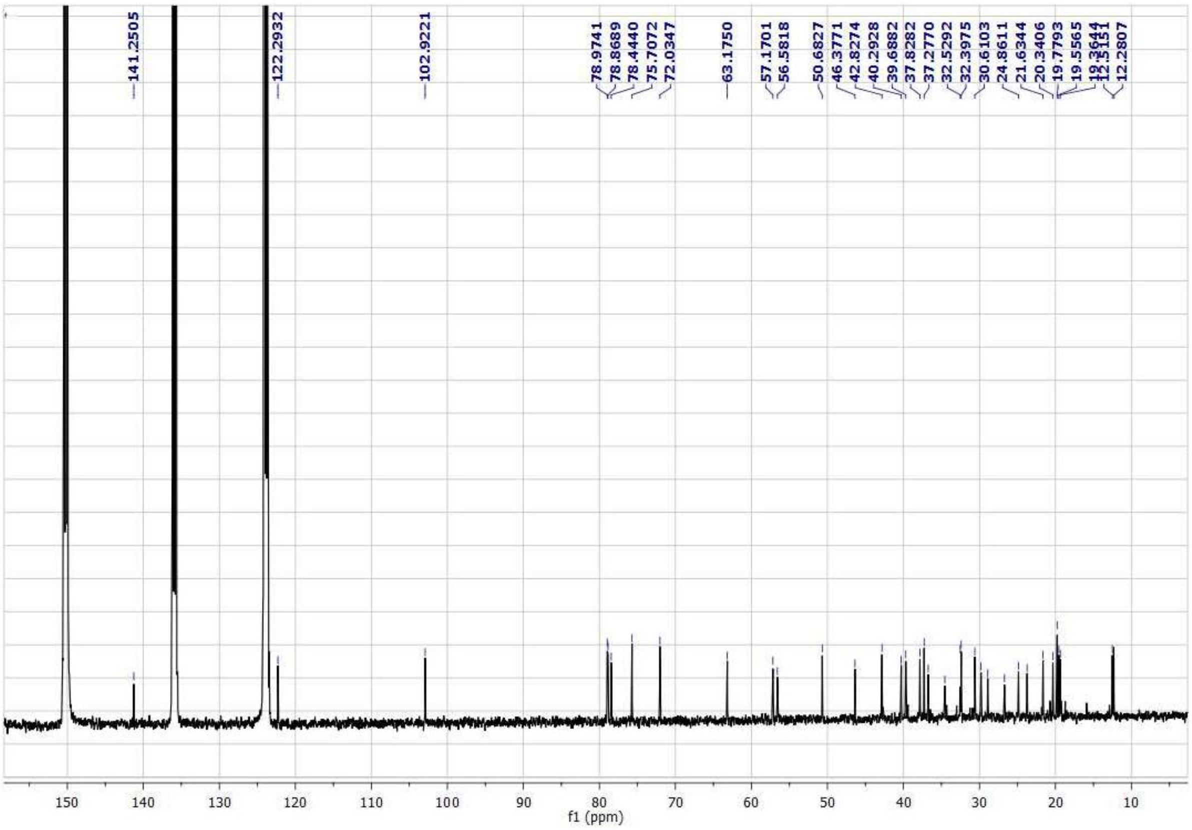

Figure S2: ¹³C NMR (125 MHz) spectrum of *β*-sitosterol-3-*O*-*β*-D-glucoside (1) in C_5D_5N

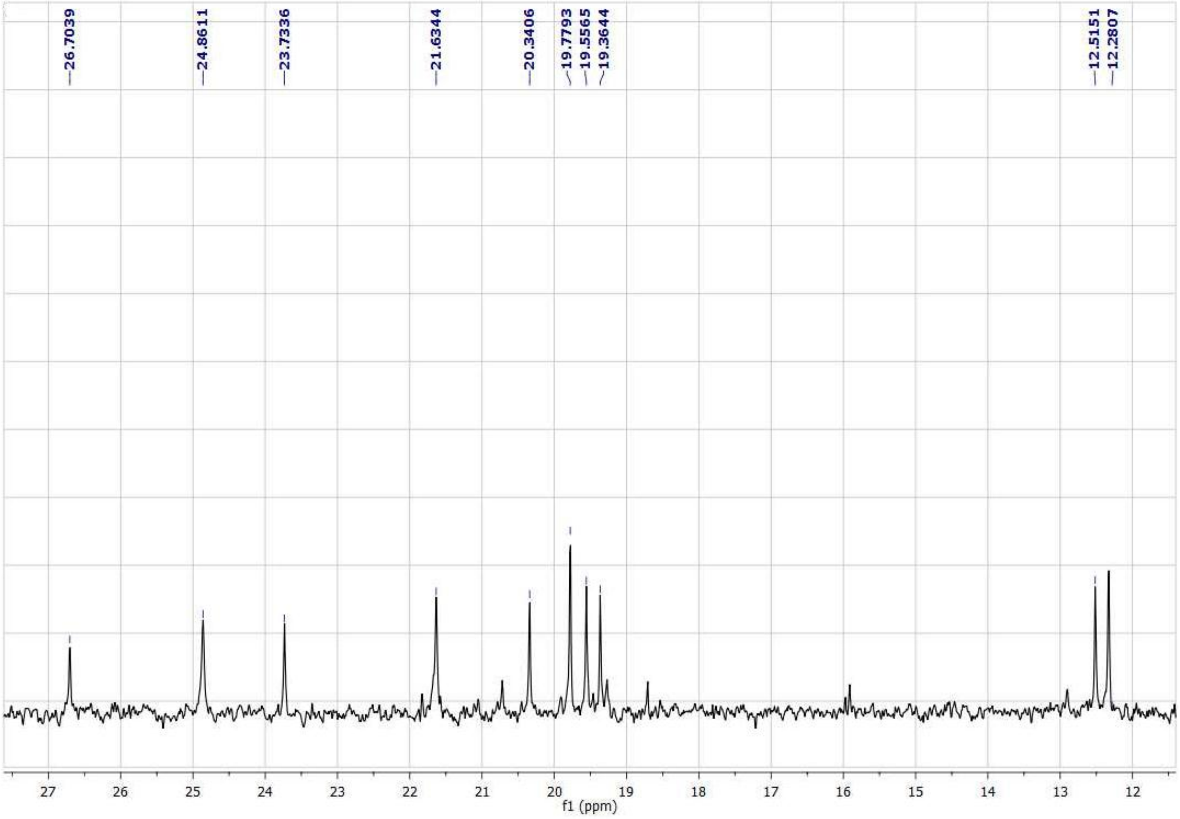

Figure S2a: Expanded ^{13}C NMR (125 MHz) spectrum of *β*-sitosterol-3-*O*-*β*-D-glucoside (1) in C_5D_5N

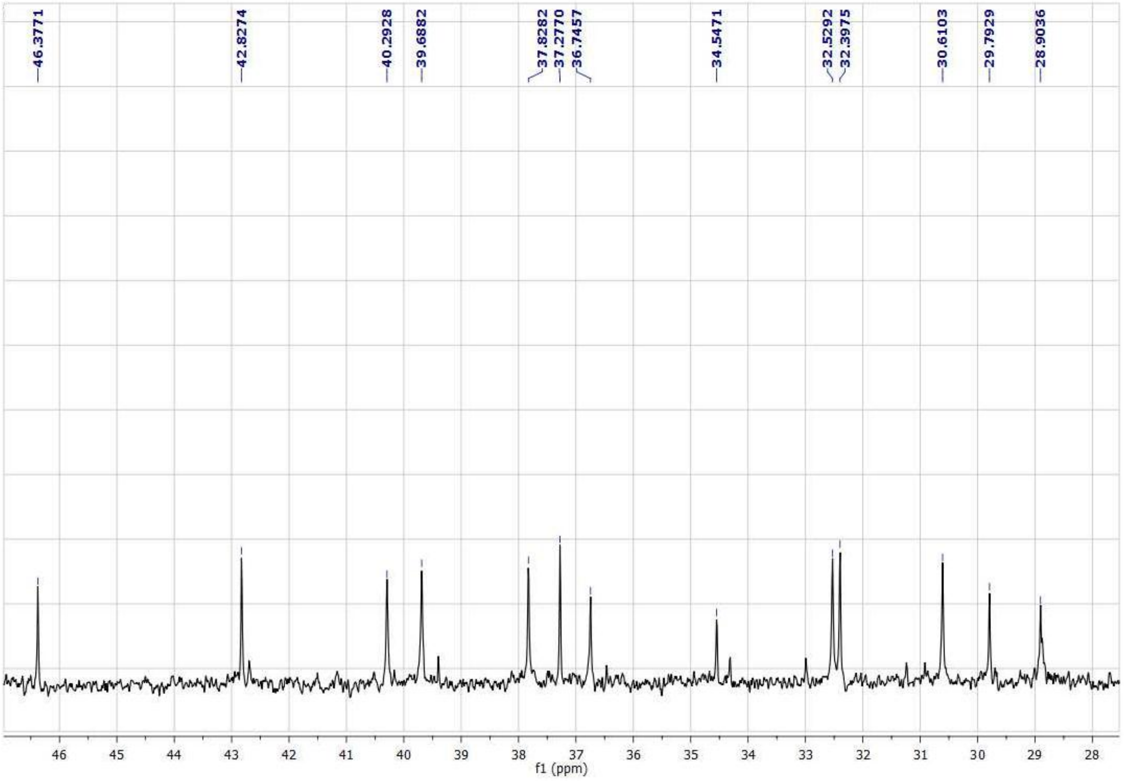

Figure S2b: Expanded ^{13}C NMR (125 MHz) spectrum of *β*-sitosterol-3-*O*-*β*-D-glucoside (1) in C_5D_5N

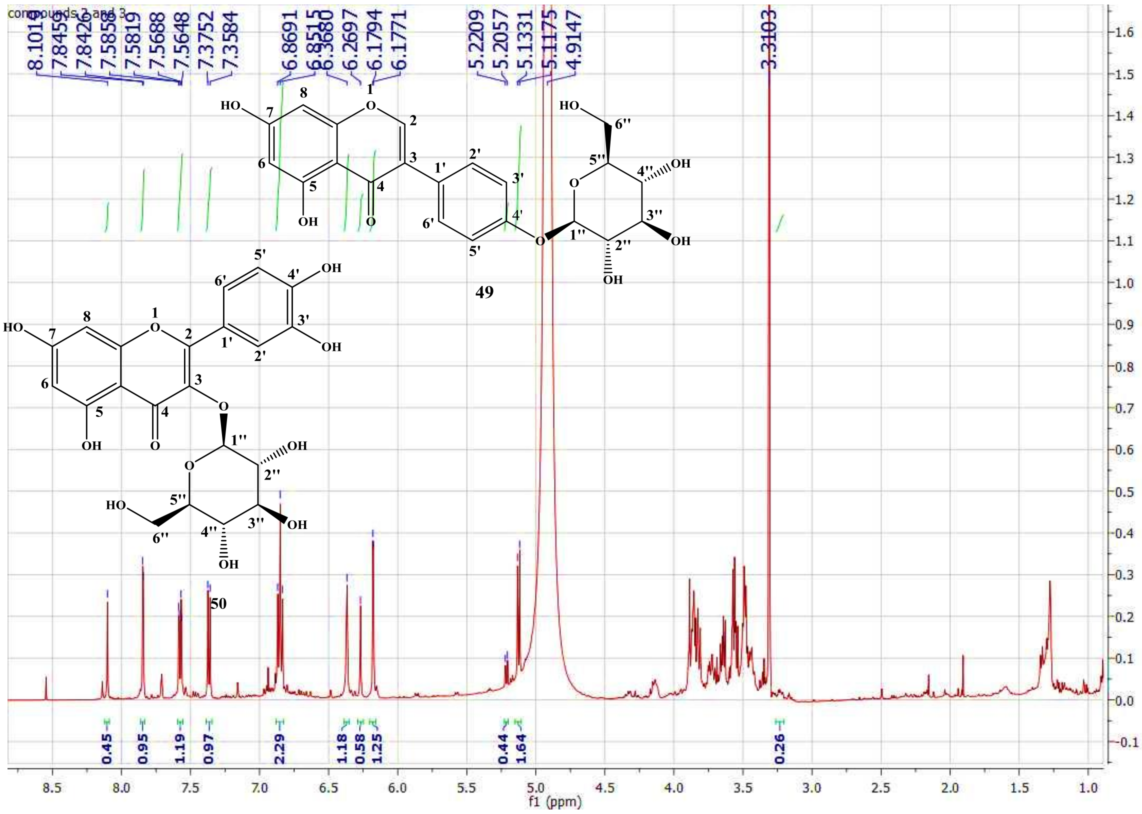

Figure S3: ^{1}H NMR (500 MHz) spectrum of genistein-4'-*β*-*O*-D-glucopyranoside (2) and quercetin-3-*β*-*O*-D-glucoside (3) in CD_3OD

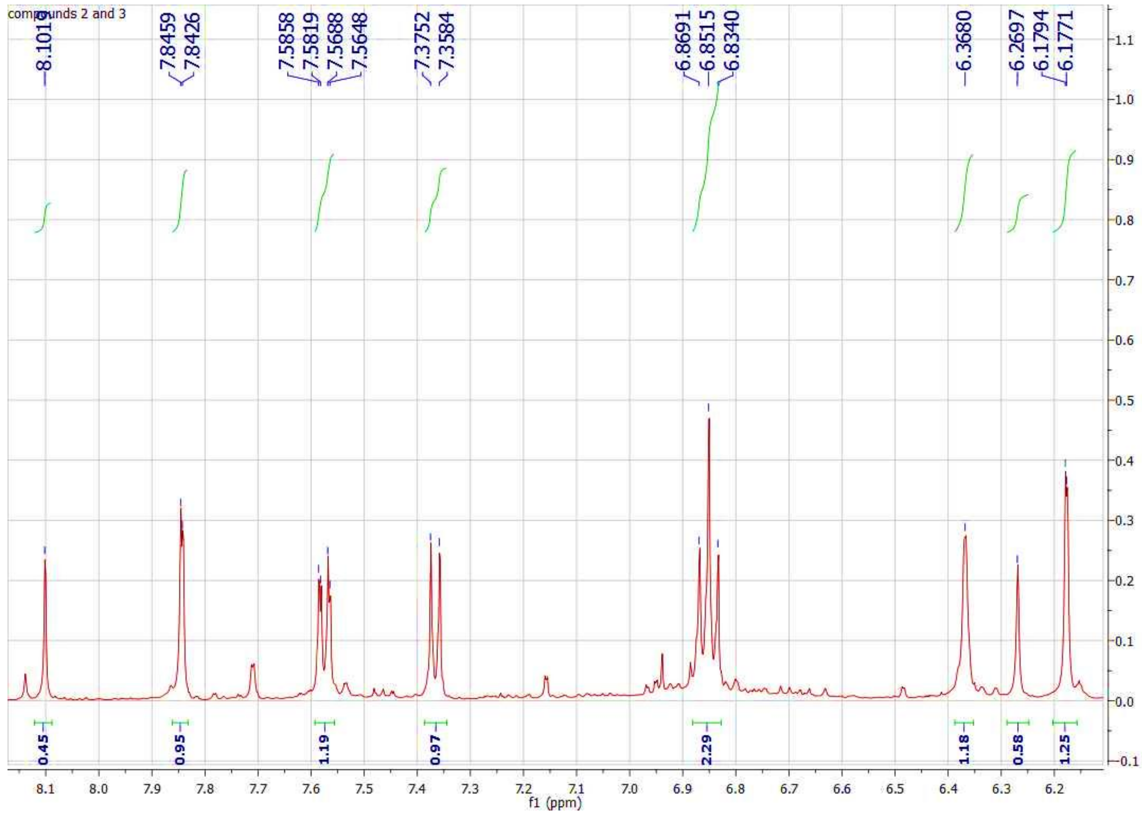

Figure S3a: Expanded ^{1}H NMR (500 MHz) spectrum of genistein-4'-*β*-*O*-D-glucopyranoside (2) and quercetin-3-*β*-*O*-D-glucoside (3) in CD_3OD

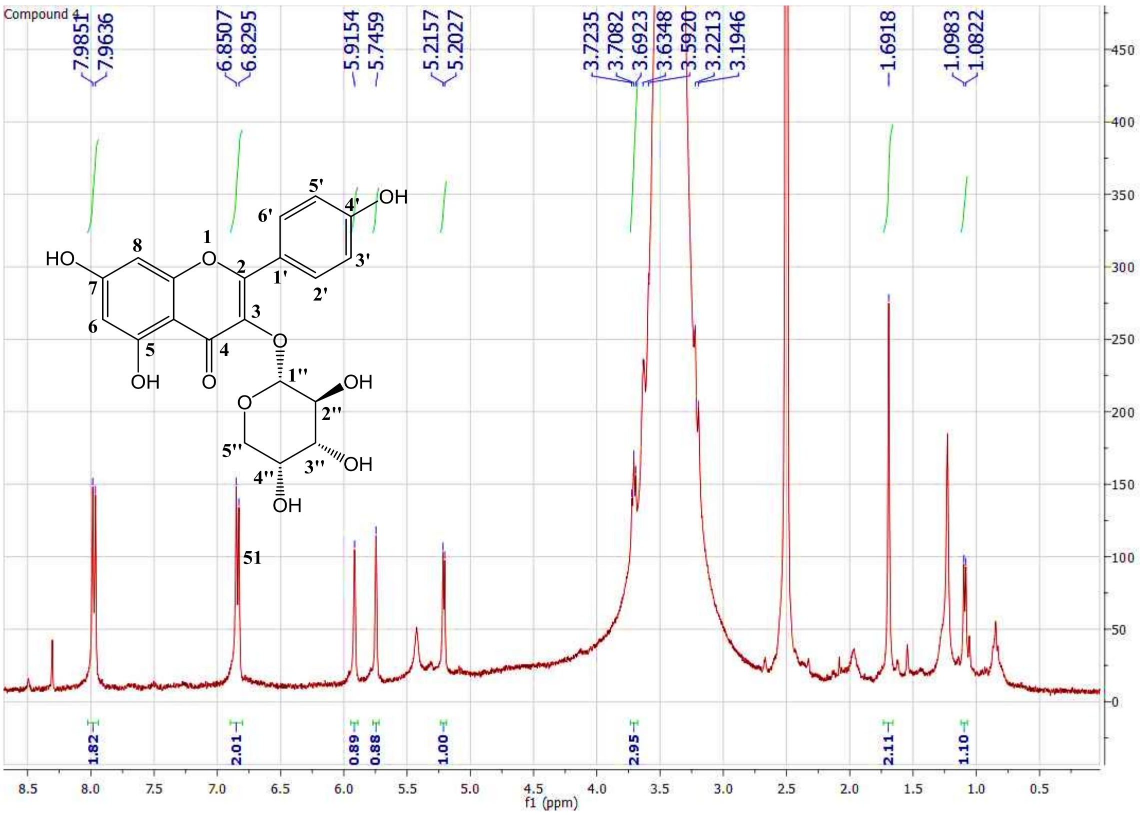

Figure S4: ^{1}H NMR (500 MHz) spectrum of kaempferol-3-*O*-*α*-D-arabinopyranoside (4) in DMSO-*d6*

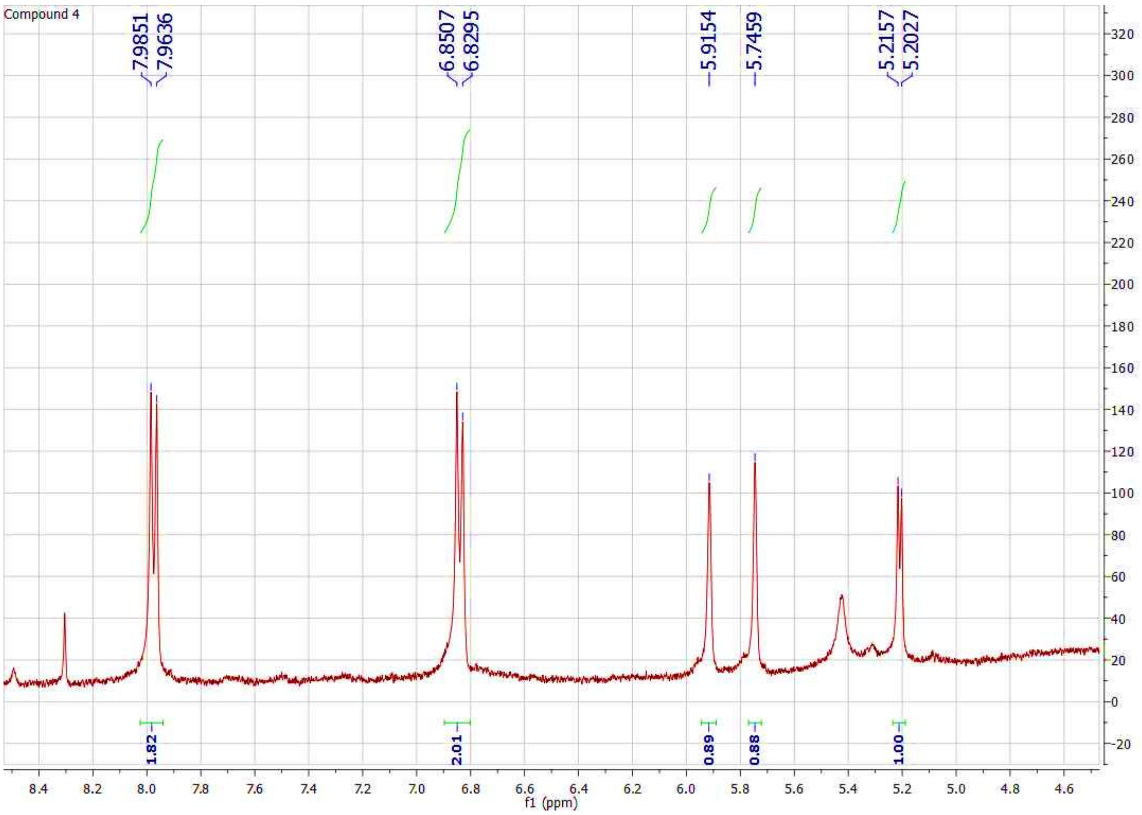

Figure S4a: Expanded ^{1}H NMR (500 MHz) spectrum of kaempferol-3-*O*-*α*-D-arabinopyranoside (4) in DMSO-*d6*

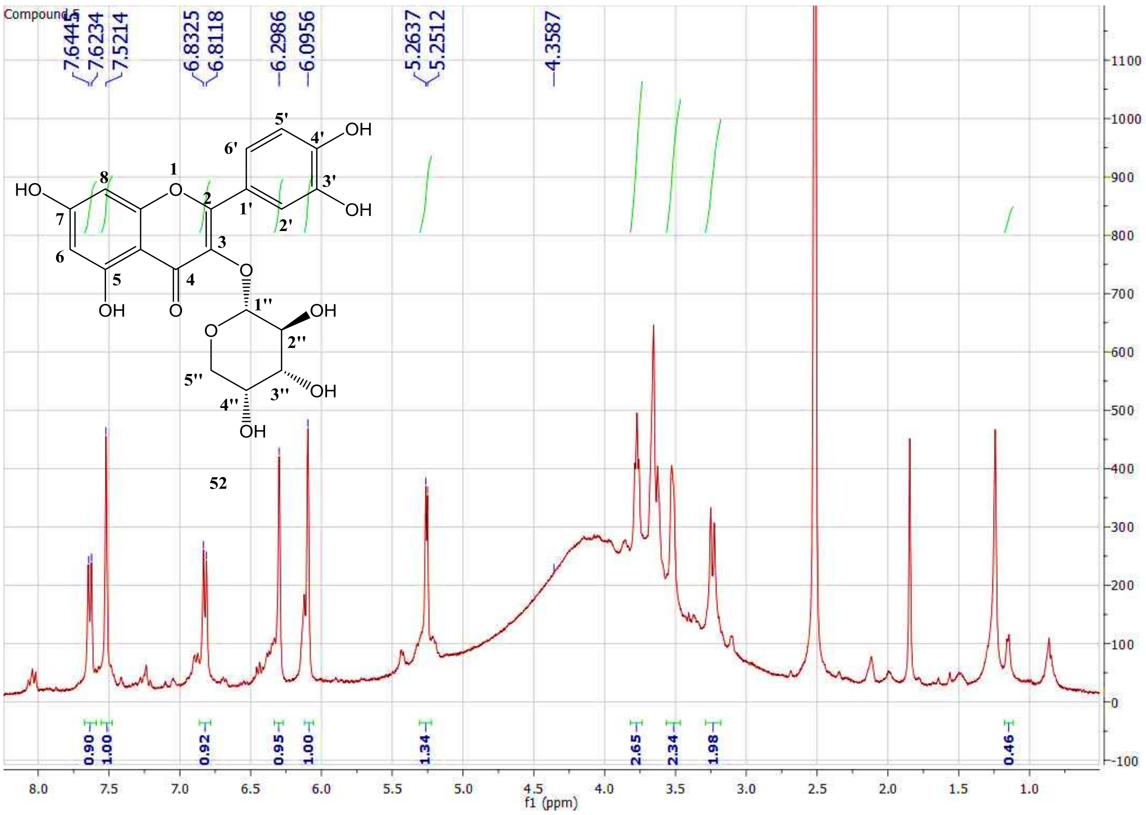

Figure S5: ^{1}H NMR (500 MHz) spectrum of quercetin-3-*O*-*α*-D-arabinopyranoside (5) in DMSO-*d6*

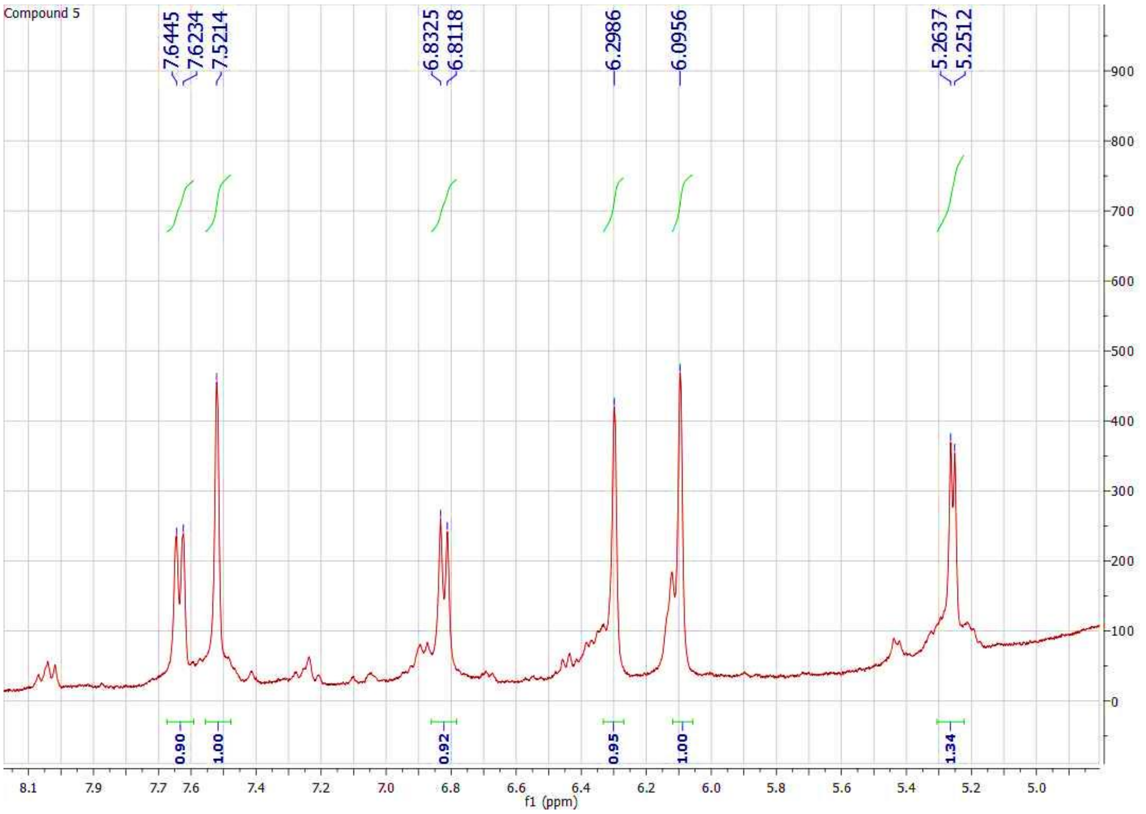

Figure S5a: Expanded ^{1}H NMR (500 MHz) spectrum of quercetin-3-*O*-*α*-D-arabinopyranoside (5) in DMSO-*d6*

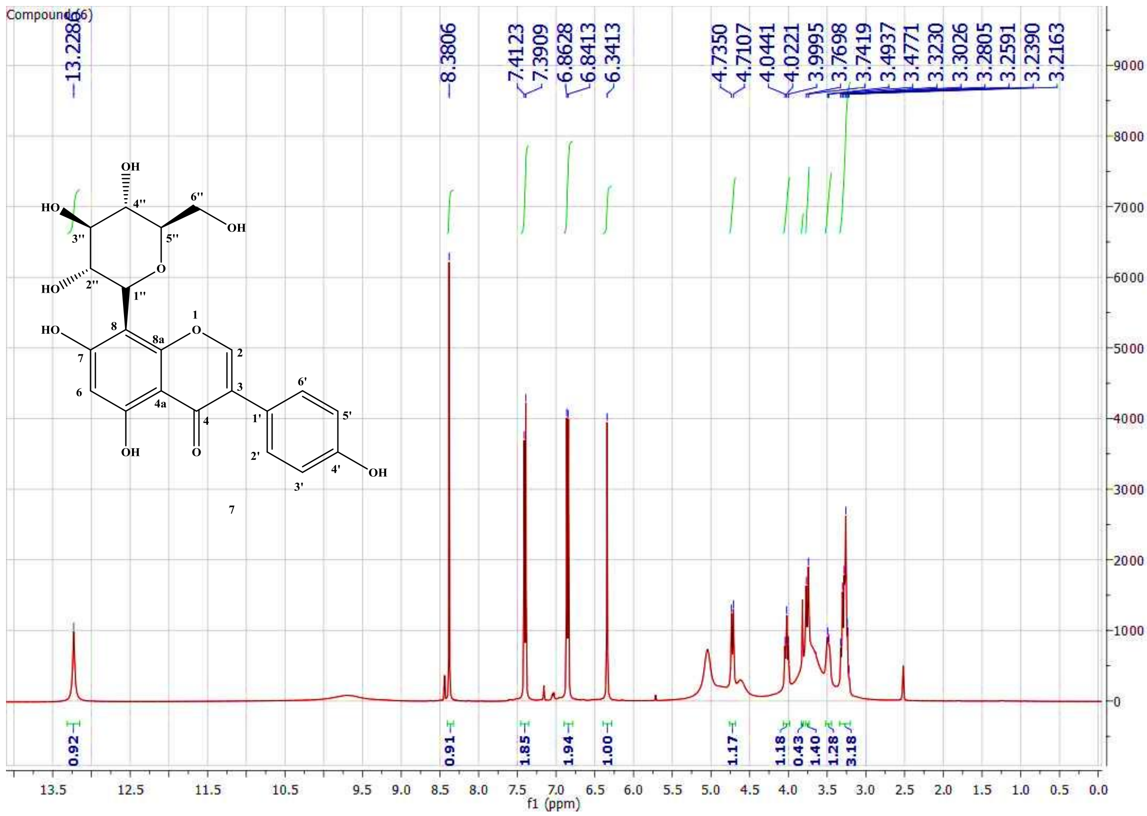

Figure S6: ^{1}H NMR (400 MHz) spectrum of genistein-8*β*-*C*-glucoside (6) in DMSO-*d*6

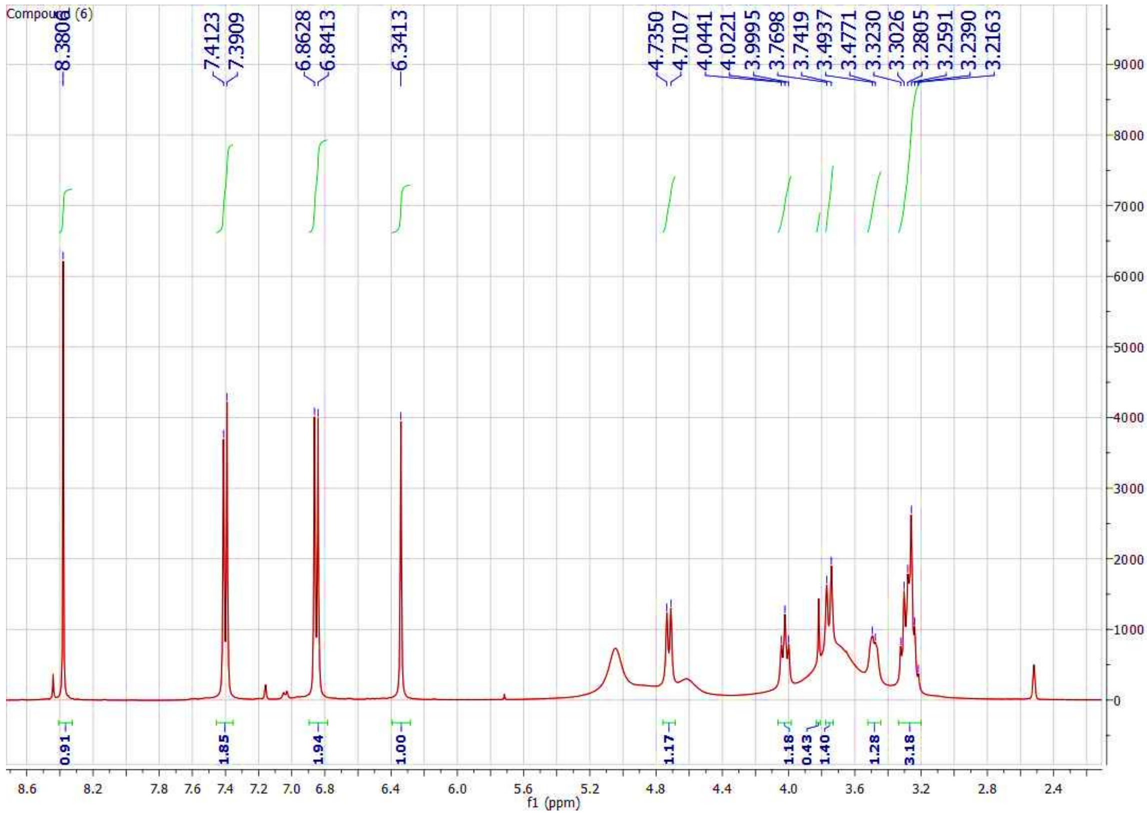

Figure S6a: Expanded ^{1}H NMR (400 MHz) spectrum of genistein-8*β*-*C*-glucoside (6) in DMSO-*d*6

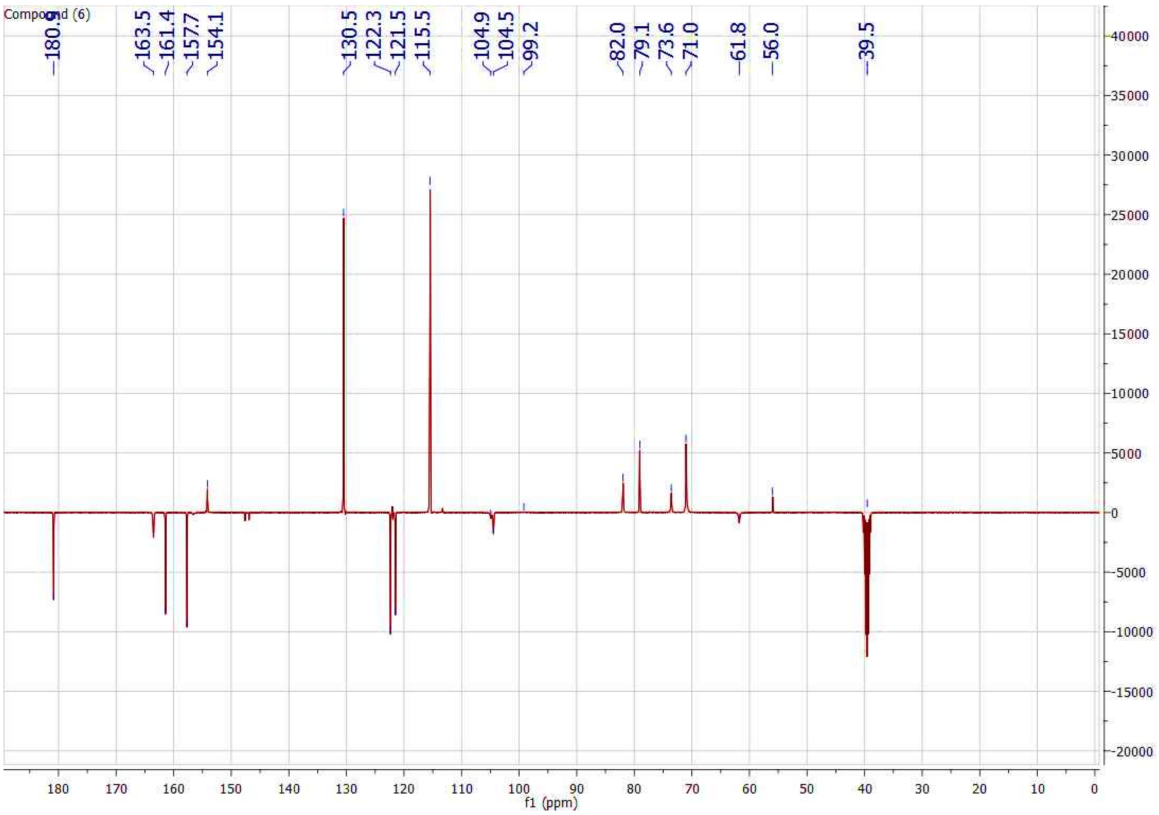

Figure S7: APT ^{13}C NMR (100 MHz) spectrum of genistein-8*β*-C-glucoside (6) in DMSO-*d6*

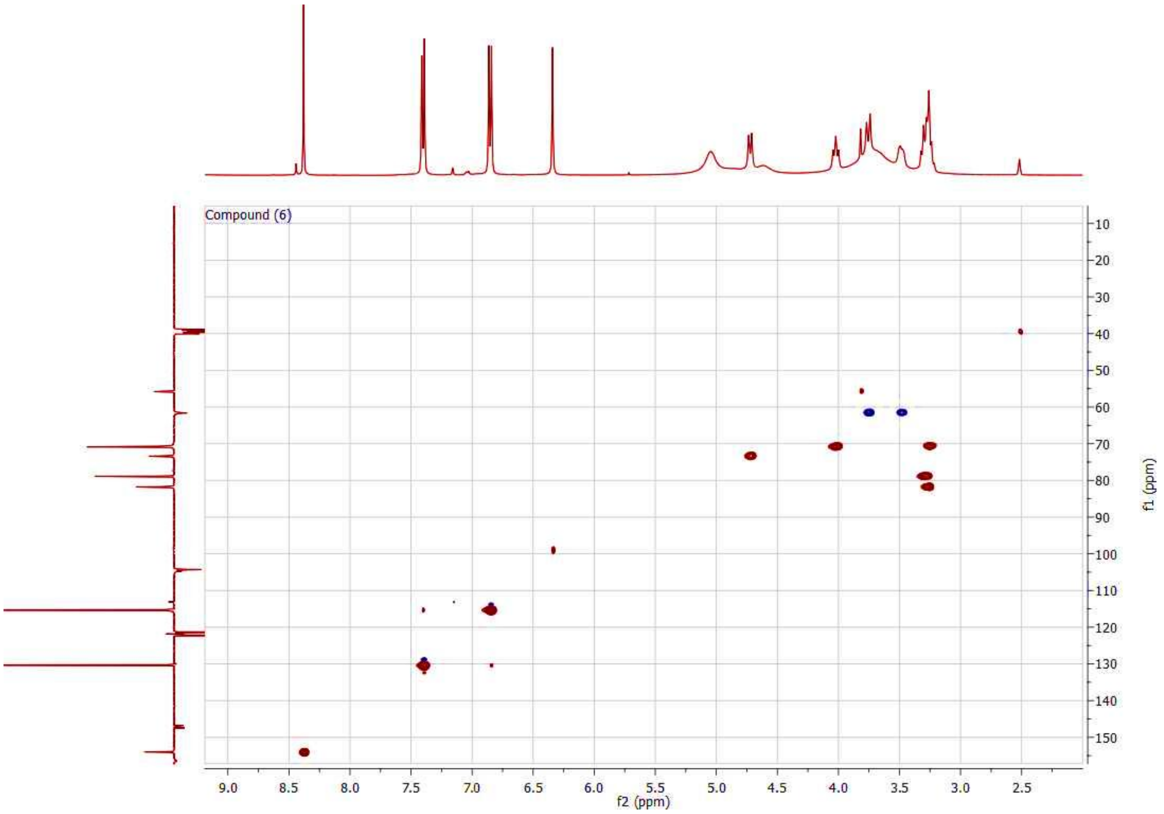

Figure S8: Edited HSQC spectrum of genistein-8*β*-C-glucoside (6) in DMSO-*d*6 in DMSO-*d6*

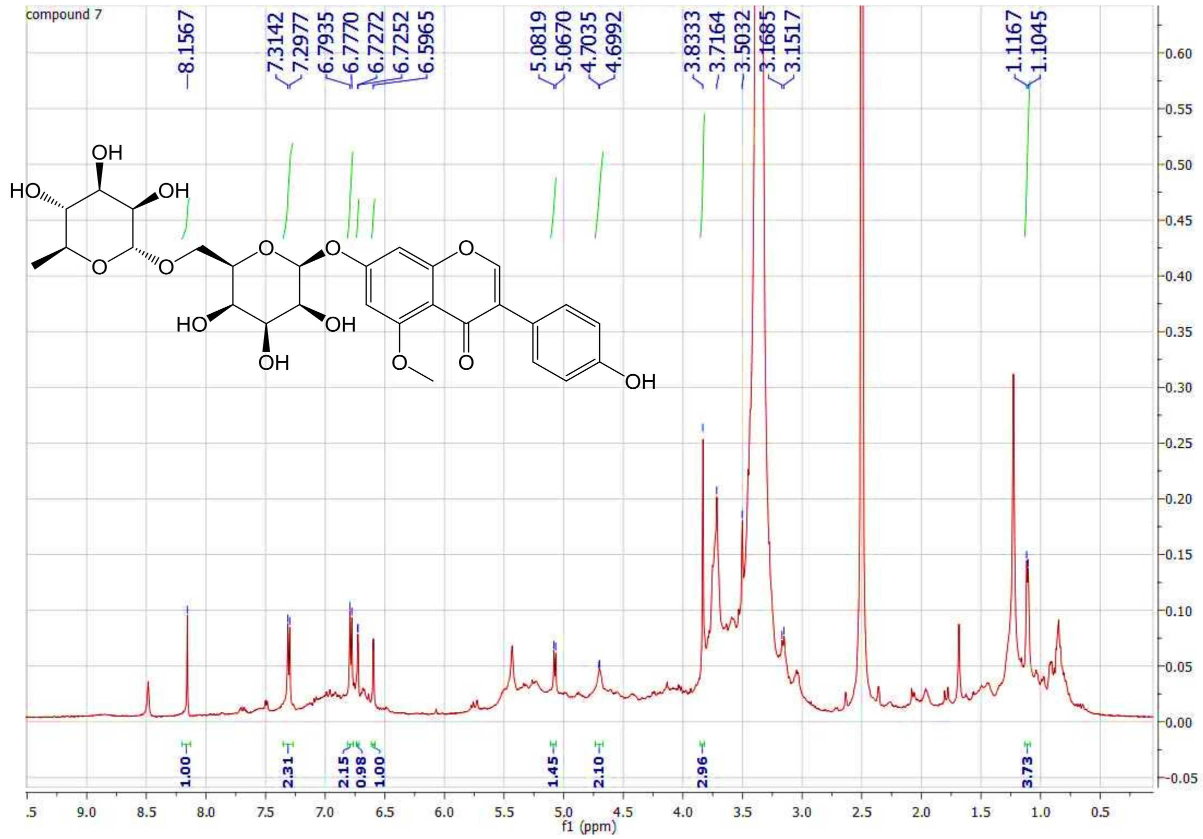

Figure S9: ^{1}H NMR (500 MHz) spectrum of hirsutissimiside B (7) in CD_3OD

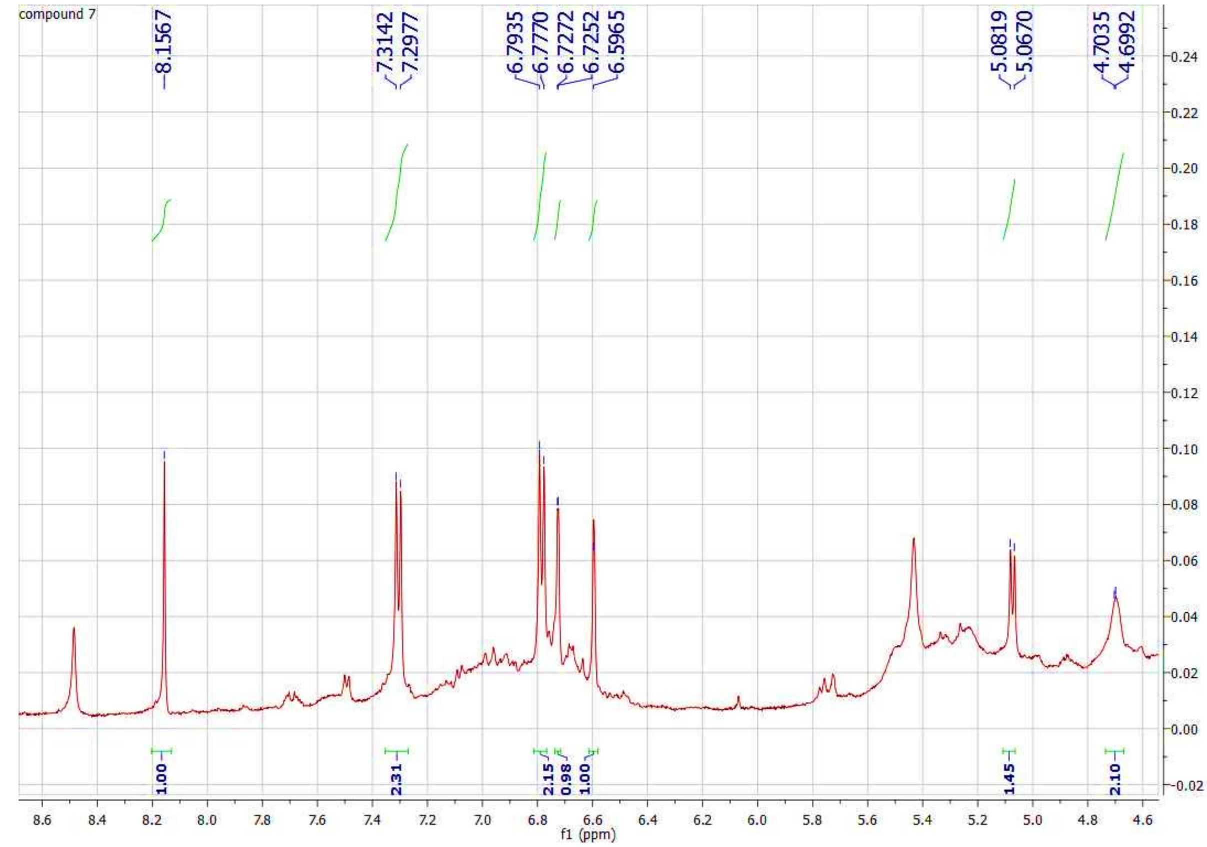

Figure S9a: Expanded ^{1}H NMR (500 MHz) spectrum of hirsutissimiside B (7) in CD_3OD
